# Supplementary figures and images for: A Sinorhizobium meliloti and Agrobacterium tumefaciens ExoR ortholog is not crucial for Brucella abortus virulence
Source: PLoS One. 2021 Aug 13;16(8):e0254568. doi: 10.1371/journal.pone.0254568 (PMC8362948; doi:10.1371/journal.pone.0254568)

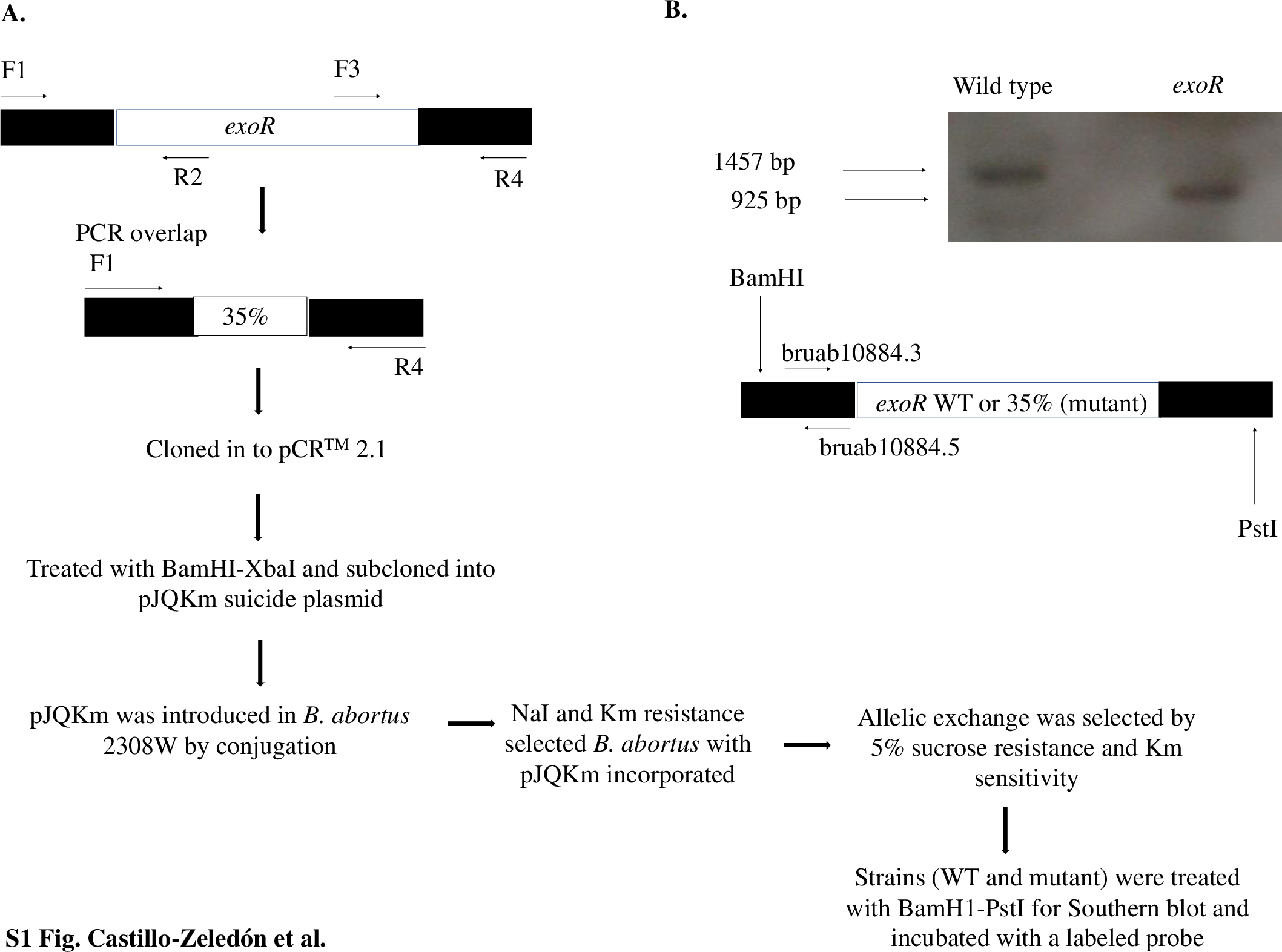

Supplement: S1 Fig — (A) Schematic representation of the in-frame deletion strategy used to construct the exoR mutant. Primers exoR-F1 and exoR-R2 were used to generate fragment 1, and fragment 2 was generated using exoR-F3, and exoR-R4 (S1 Table). Both fragments were ligated by PCR overlapping using nucleotides exoR-F1 and exoR-R4. The resulting deletion allele was cloned in the pCR™ 2.1 vector (Invitrogen™) and subcloned into the BamHI-XBaI site of the suicide plasmid pJQKm. Plasmid pJQKm containing the deleted allele was introduced in B. abortus 2308W by conjugation. Colonies corresponding to integrating the suicide vector in the chromosome were selected using Nalidixic acid (NaI, 25 μg/mL) and Kanamycin (Km, 50 μg/mL) resistance. Excision of the suicide plasmid leading to the mutant’s construction by allelic exchange was selected by 5% sucrose resistance and Km sensitivity. The resulting colonies were screened using primers exoR-F1 and exoR-R4. Mutant colonies generated a 780 bp fragment and the parental strain a 1200 bp fragment. (B) Southern blot analysis. BamH1 and PstI restriction sites were chosen according to the B. abortus 2308W genome sequence, at positions -3009 and +1552, respectively, from exoR first codon. The resulting fragments were separated in a 0.7% agarose gel. After electrophoresis, the protocol was carried out with minor variations as described in Methods. Briefly, the gel was rinsed in 0.25 M HCL, denatured (1.5 M NaCl, 0.5 M NaOH), and neutralized twice (1.5 M NaCl, 0.5M Tris HCl pH8). The DNA was then transferred to a nylon membrane (Roche) overnight in 10x SSC pH 7 (0.15 M sodium citrate, 1.5 M NaCl). Primers bruabI0884.3 and bruabI0884.5 (S1 Table) were used to generate a 295 bp amplicon from B. abortus 2308W DNA. These primers amplify a region from position 68 bp to -363 from exoR first codon. The amplicon was purified from agarose gels using QIAquick Gel Extraction Kit (Qiagen) and labeled with digoxigenin for use as a probe according to the manufacturer’s [file pone.0254568.s001.tif]

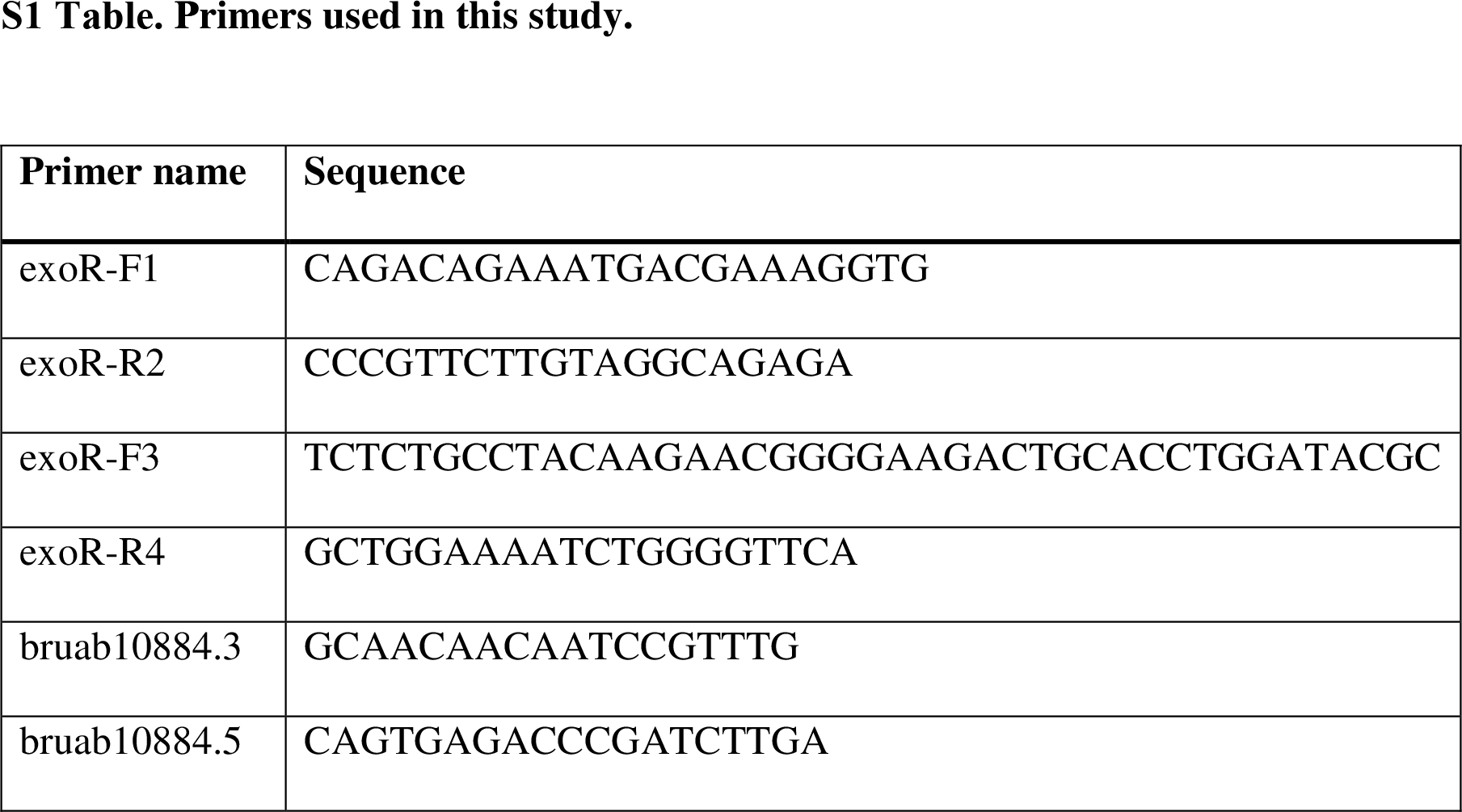

Supplement: S1 Table — (TIF) [file pone.0254568.s002.tif]
