# Supplementary material for: A Sinorhizobium meliloti and Agrobacterium tumefaciens ExoR ortholog is not crucial for Brucella abortus virulence
Source: PLoS One. 2021 Aug 13;16(8):e0254568. doi: 10.1371/journal.pone.0254568 (PMC8362948; doi:10.1371/journal.pone.0254568)

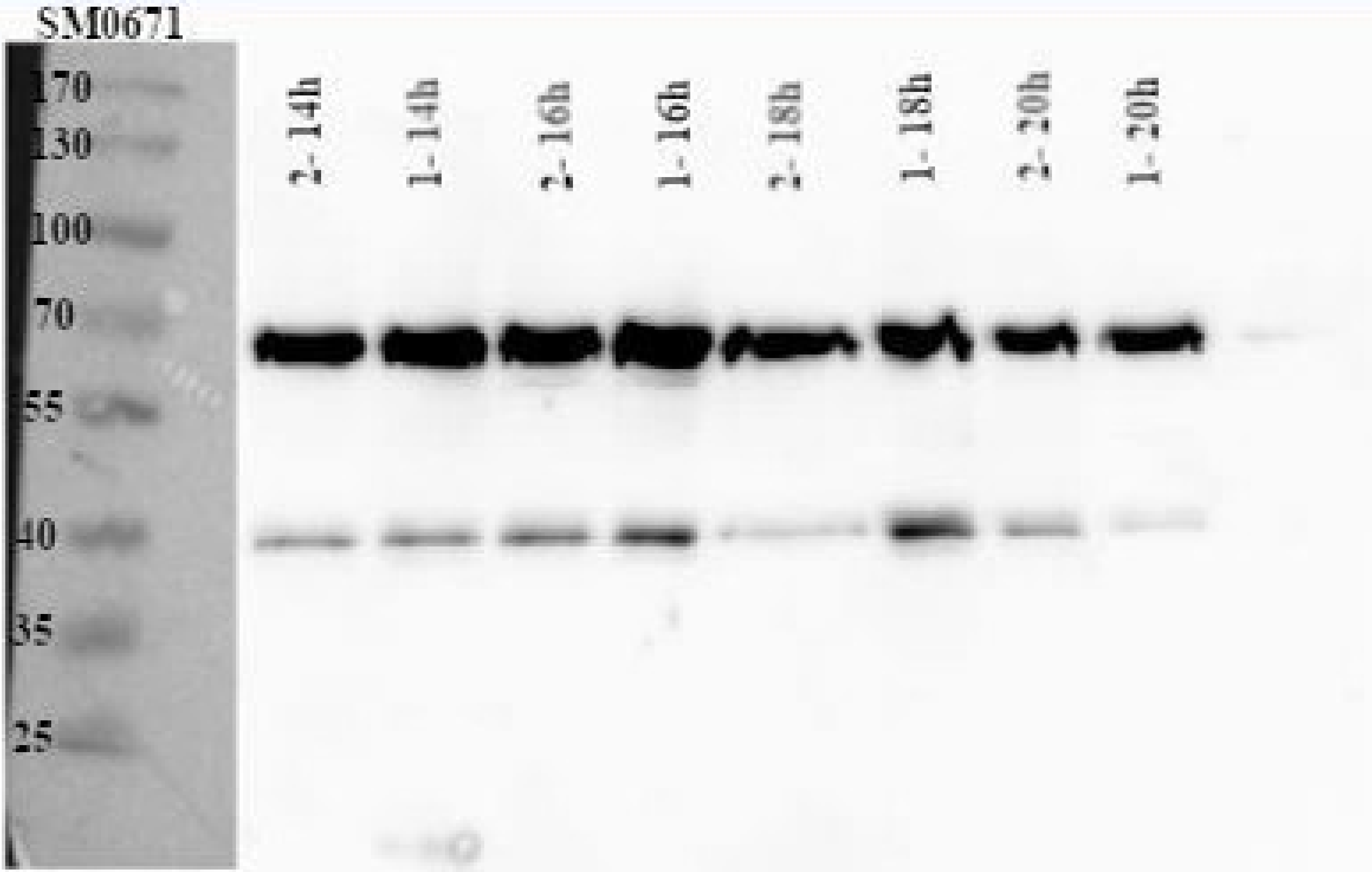

1- *exoR* mutant
2- B.a. 2308

Fig 2 BvrS
ChemiDoc Imaging Systems

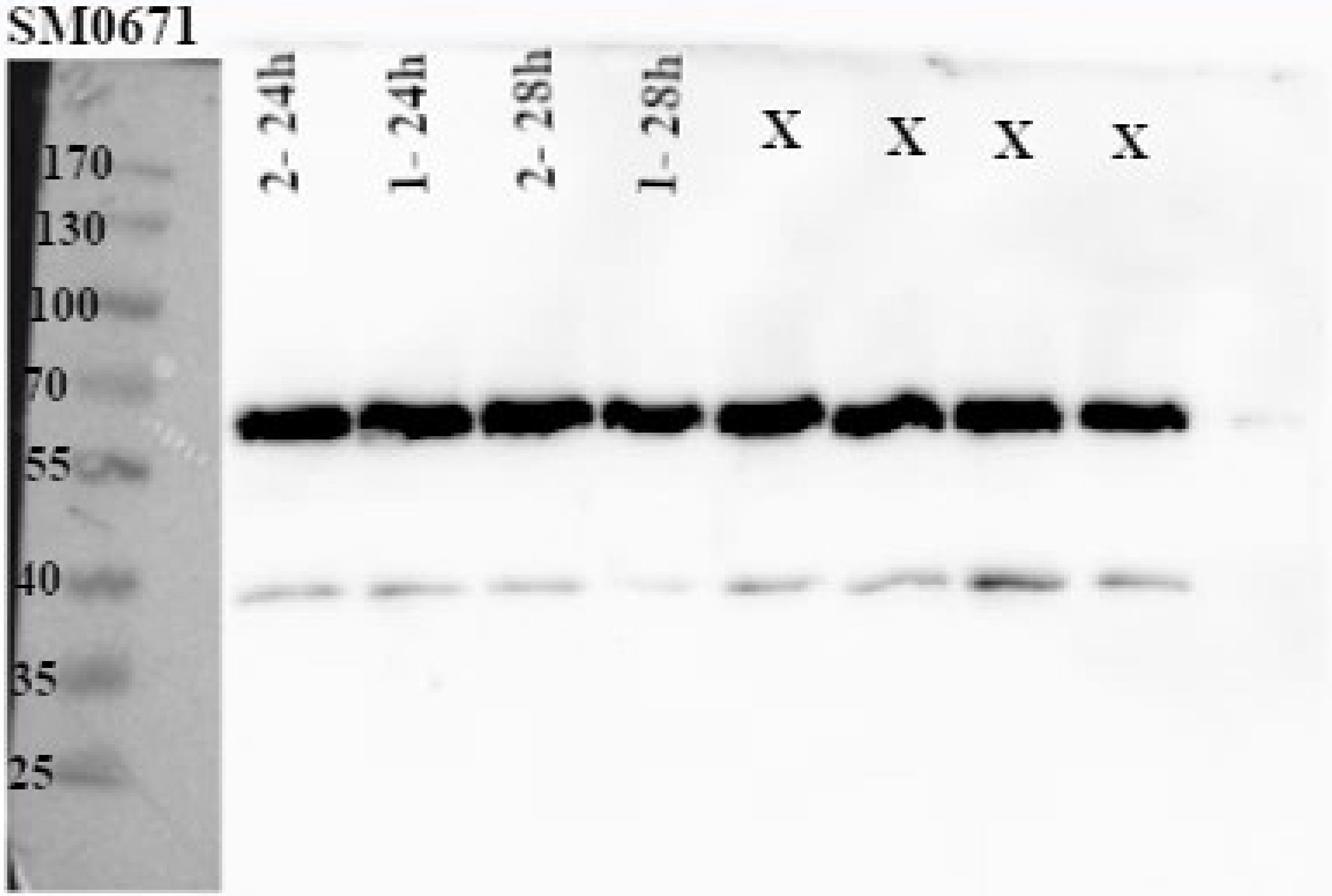

1- *exoR* mutant
2- B.a. 2308W

Fig 2 BvrS
ChemiDoc Imaging Systems Bio-Rad

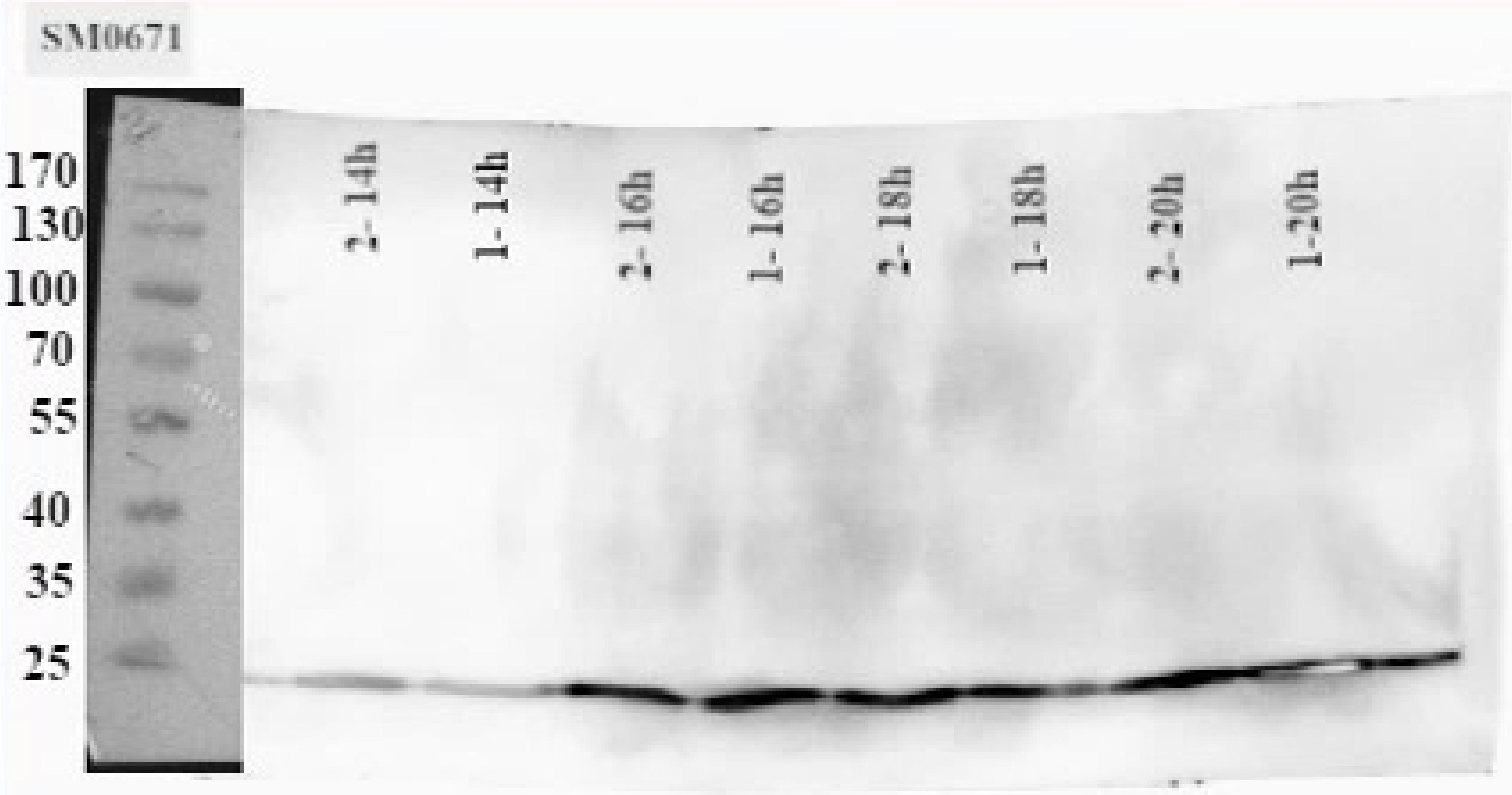

1- *evoR* mutant
2- B.a. 2308W

Omp19
Loading control for BvrS gel 1
Fig 2
ChemiDoc Imaging System Bio-Rad

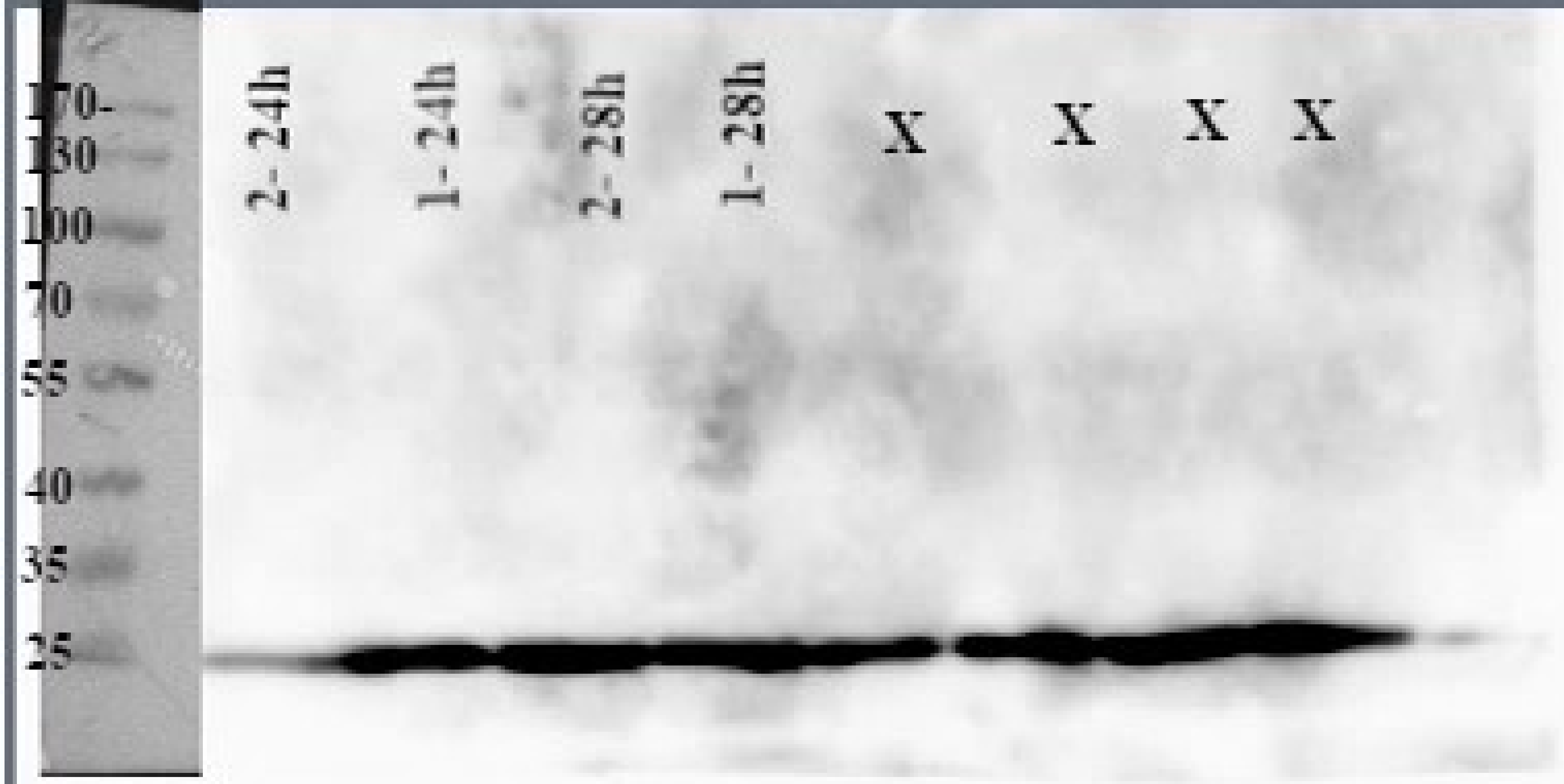

SM0671

1- *evoR* mutant
2- B.a. 2308W

Omp19
Loading control for BvrS gel 2
Fig 2
ChemiDoc Imaging System Bio-Rad

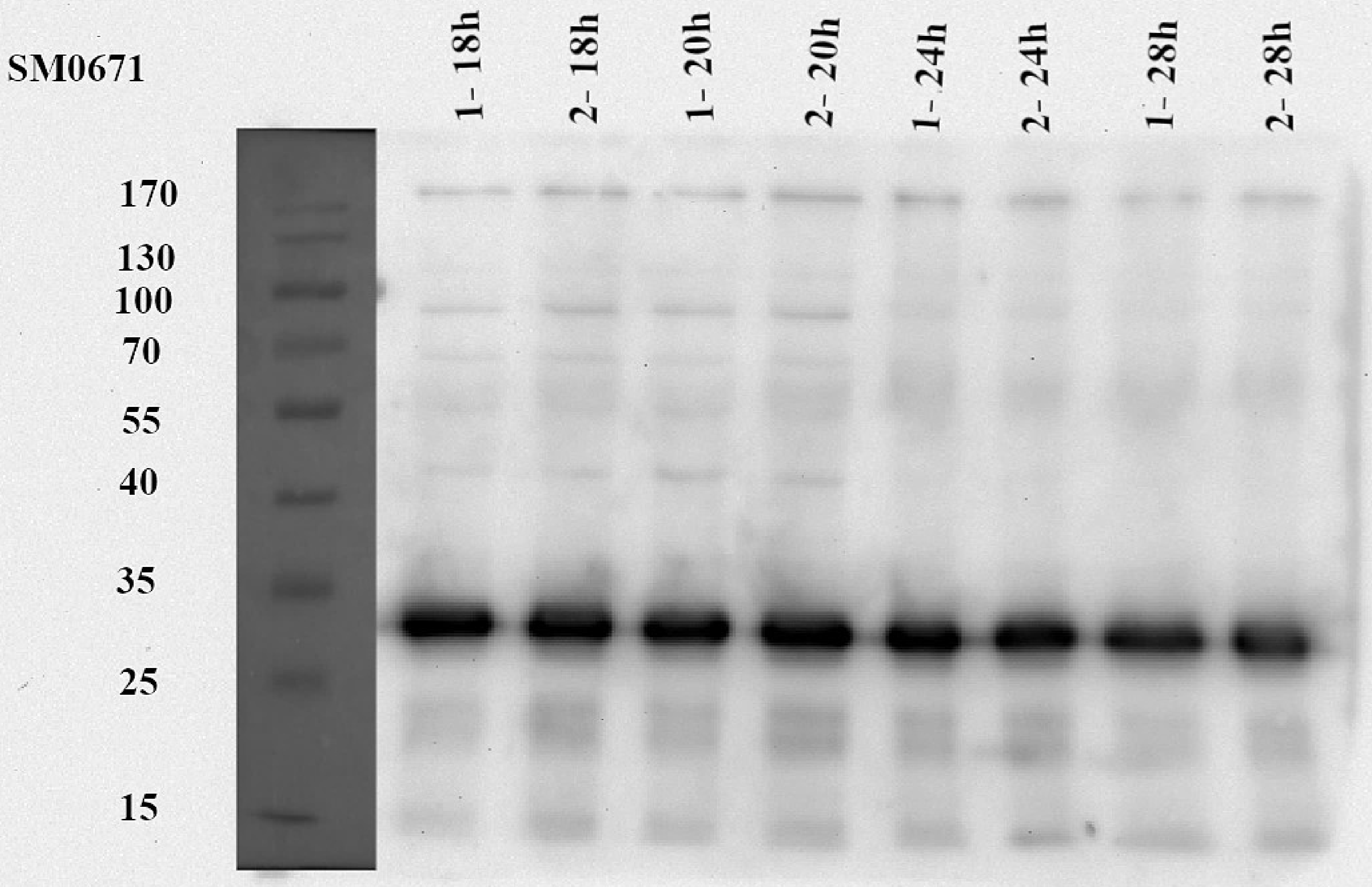

1- *exoR* mutant
2- B.a. 2308W

Fig 2 BvrR
ChemiDoc Imaging Systems Bio-Rad

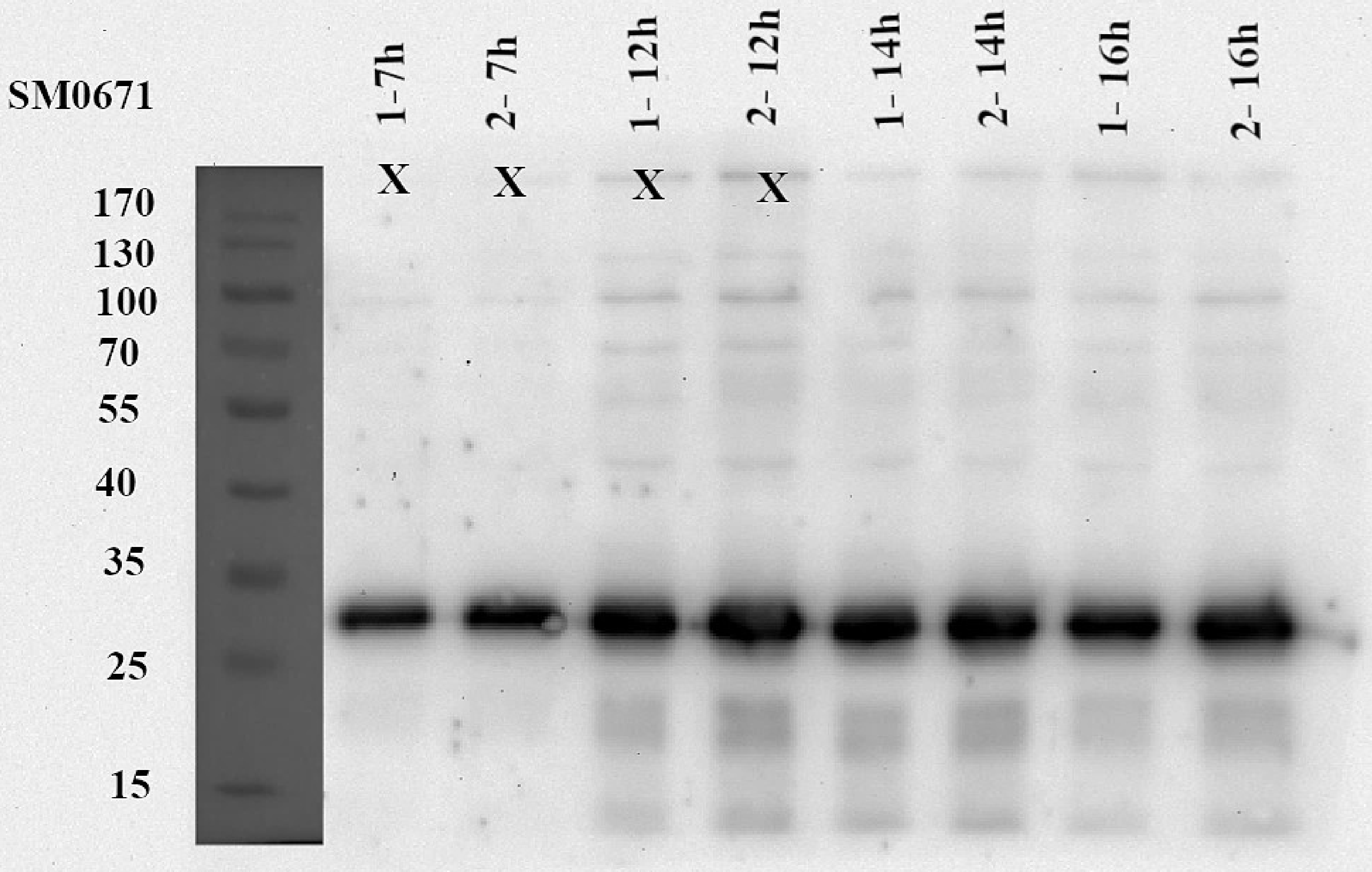

1- *exoR* mutant
2- B.a. 2308W

Fig 2 BvrR
ChemiDoc Imaging Systems Bio-Rad

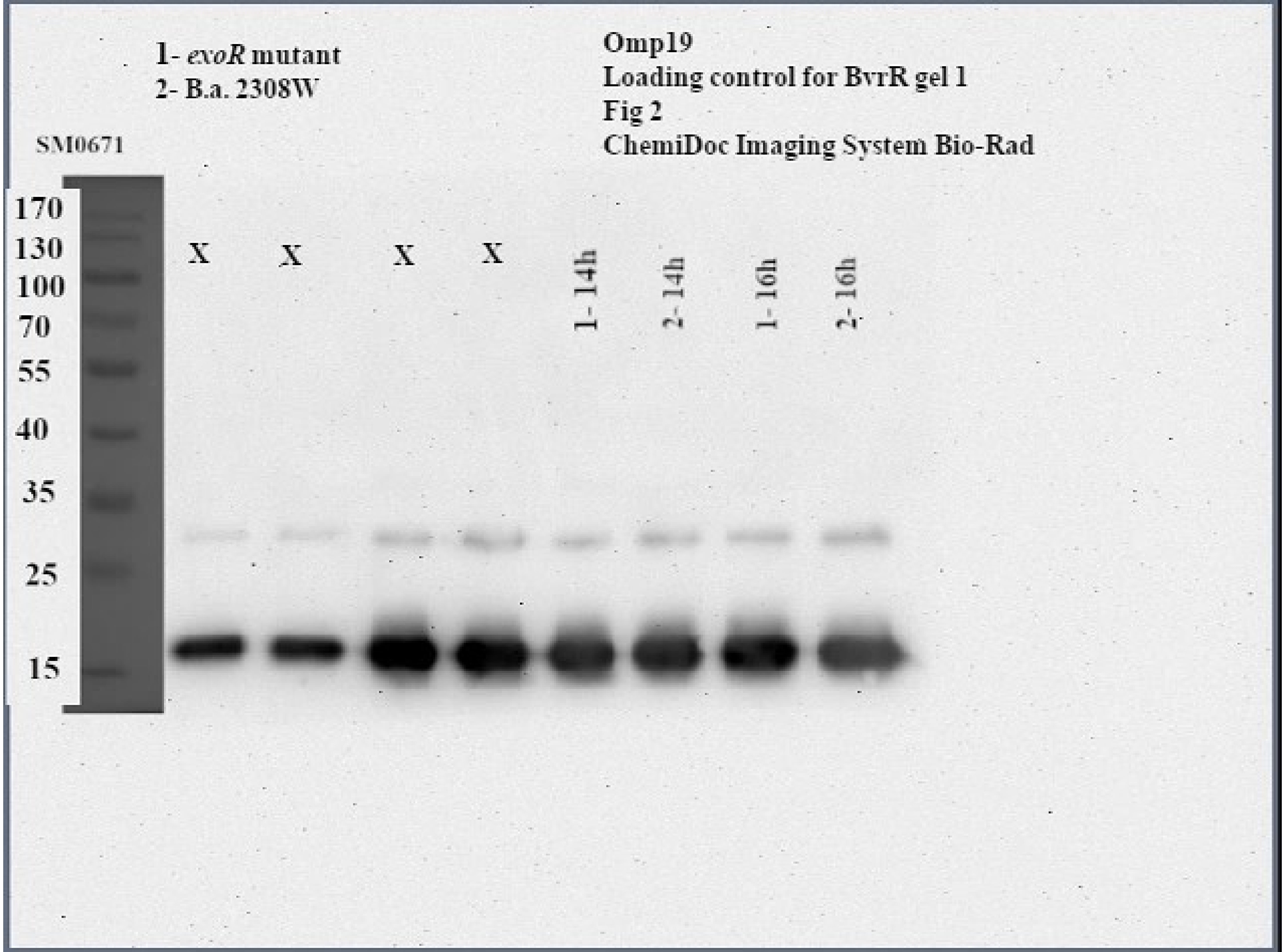
1- *exoR* mutant
2- B.a. 2308W
Omp19
Loading control for BvrR gel 1
Fig 2
ChemiDoc Imaging System Bio-Rad
SM0671
170
130
100
70
55
40
35
25
15
X
X
X
X
1- 14h
2- 14h
1- 16h
2- 16h

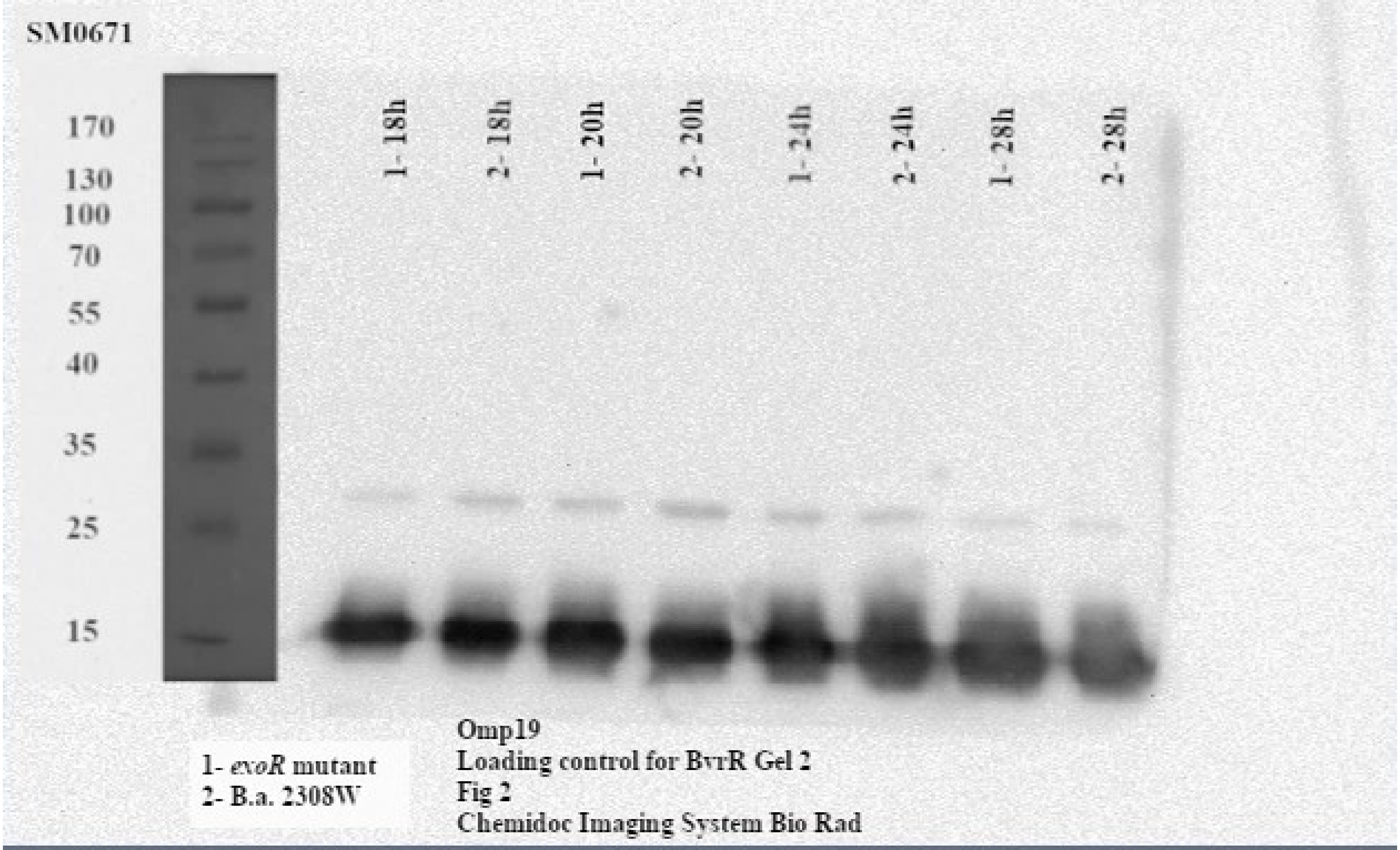
SM0671
170
130
100
70
55
40
35
25
15
1- 18h
2- 18h
1- 20h
2- 20h
1- 24h
2- 24h
1- 28h
2- 28h
1- *exoR* mutant
2- B.a. 2308W
Omp19
Loading control for BvrR Gel 2
Fig 2
Chemidoc Imaging System Bio Rad

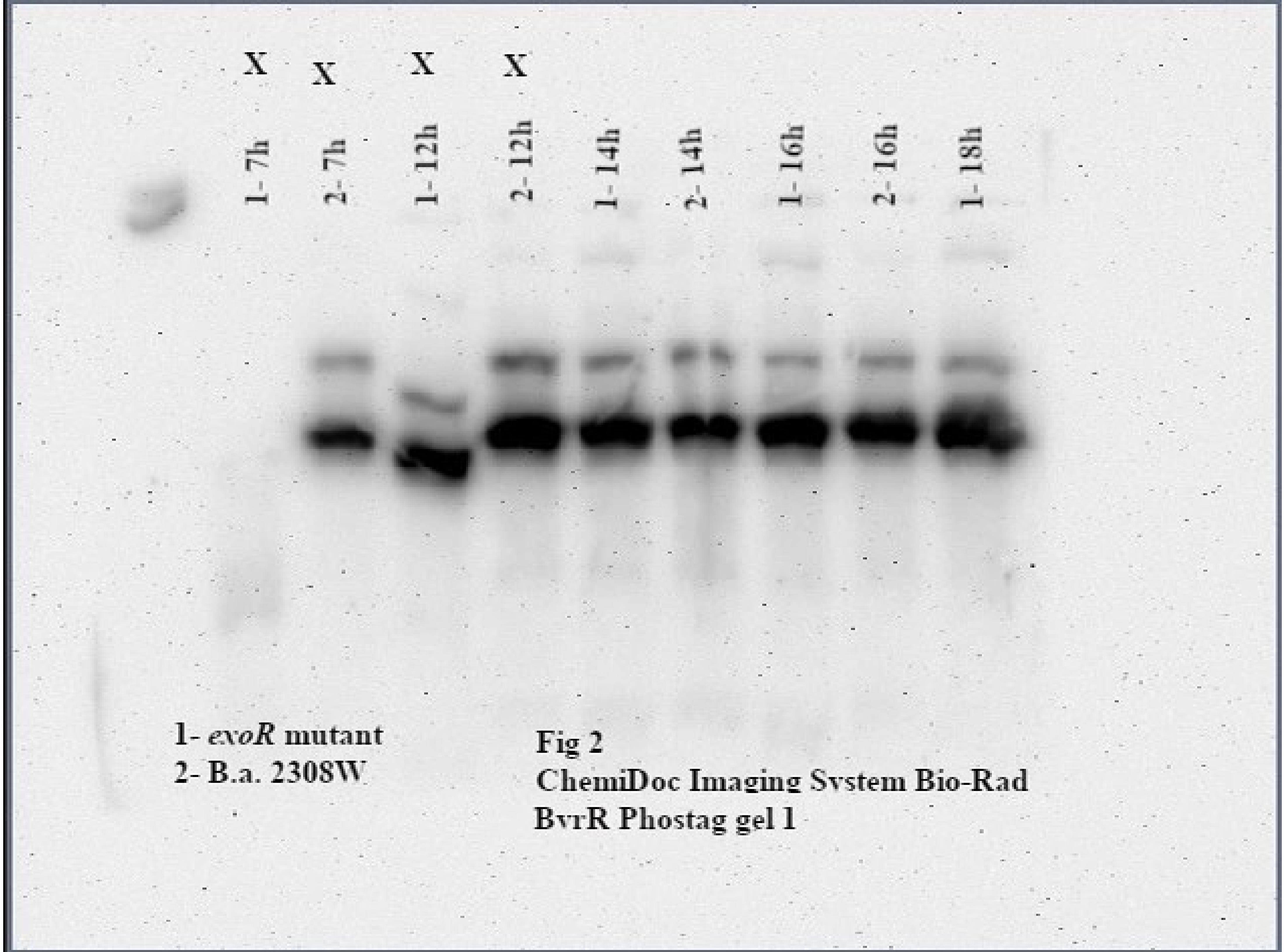

Fig 2
ChemiDoc Imaging System Bio-Rad
BvrR Phostag gel 1

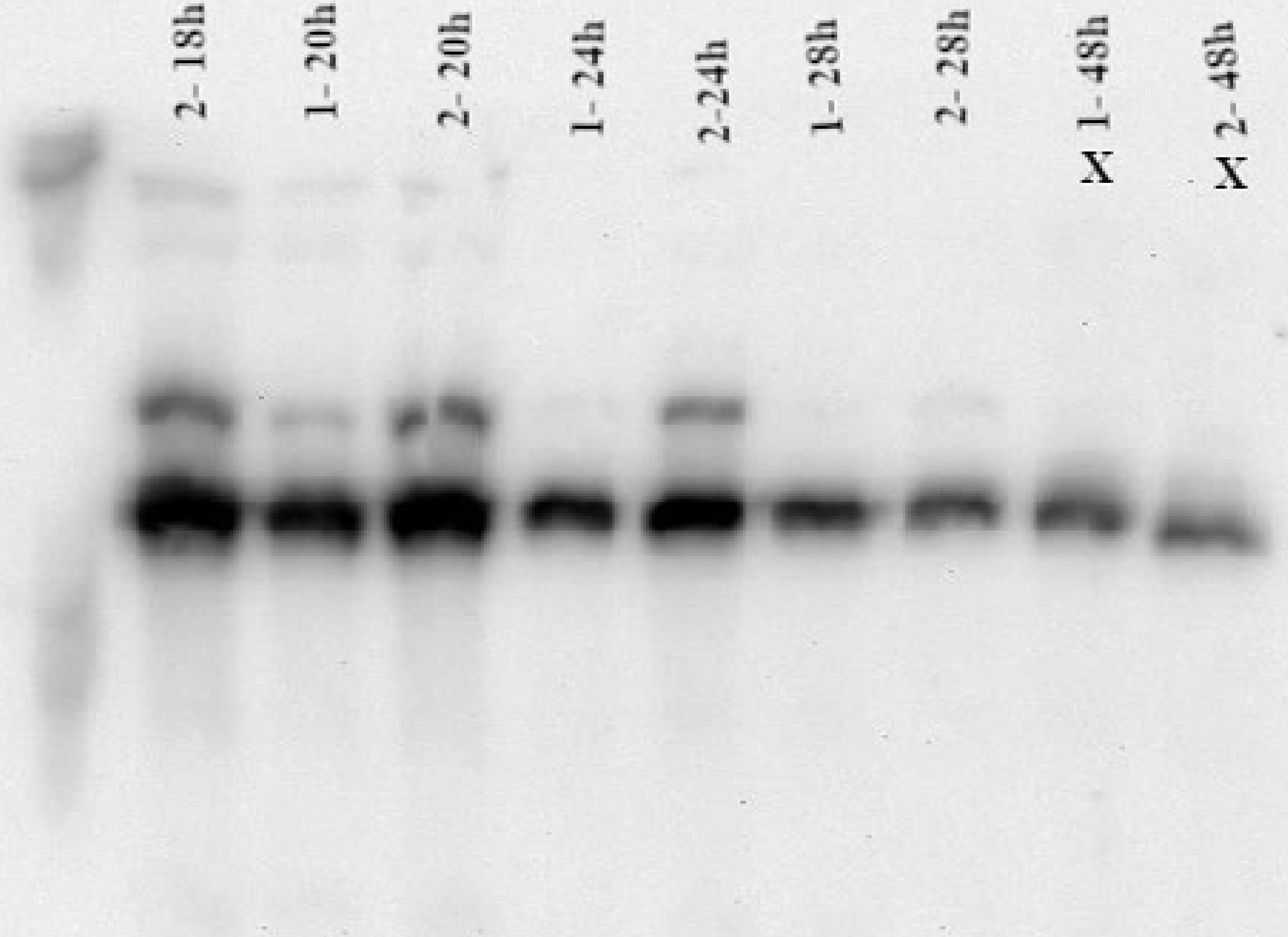

1- *exoR* mutant
2- B.a. 2308W

Fig 2 Phostag BvrR Gel 2
ChemiDoc Imaging System Bio-Rad

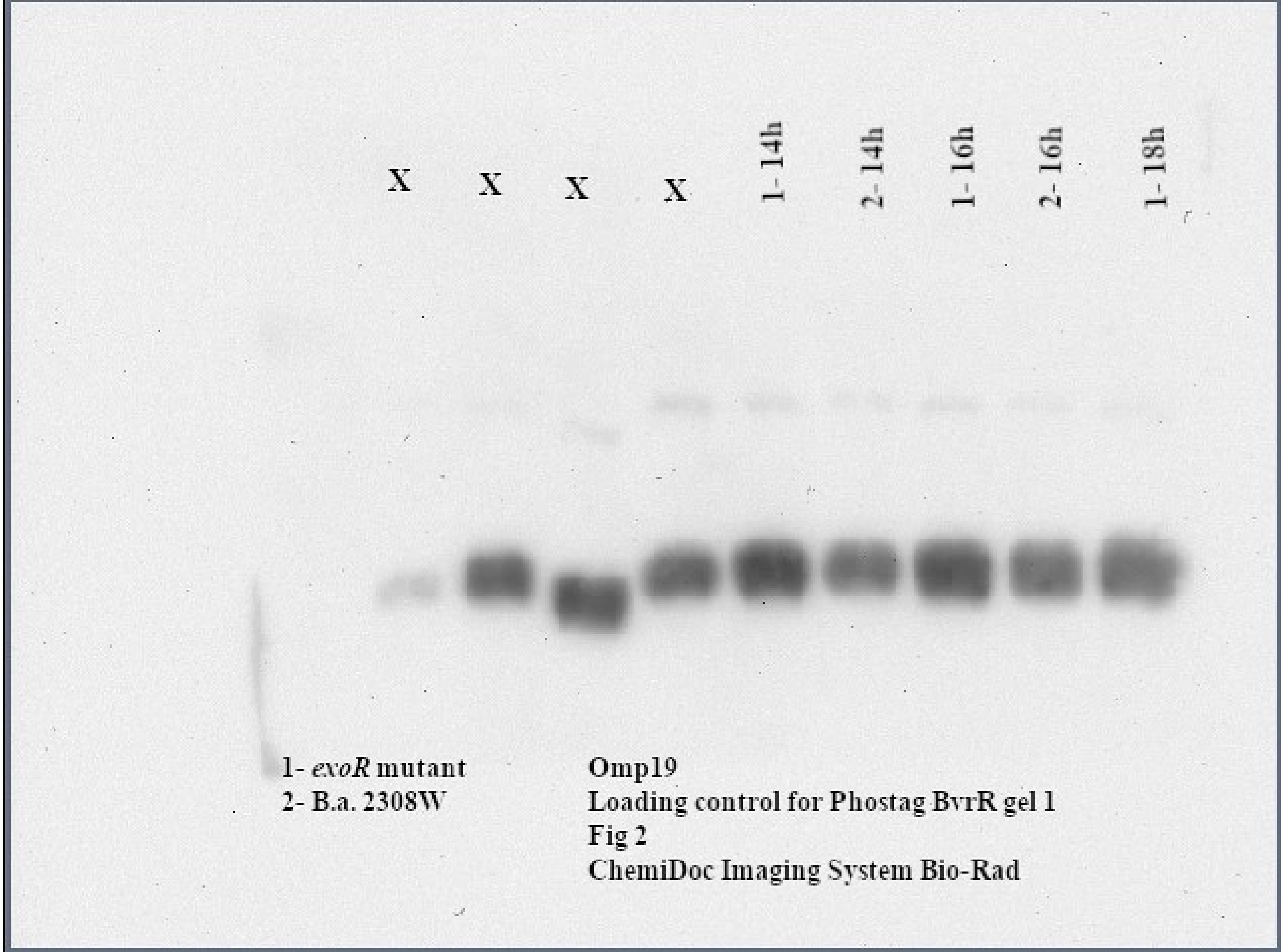
X
X
X
X
1- 14h
2- 14h
1- 16h
2- 16h
1- 18h
1- *exoR* mutant
2- B.a. 2308W
Omp19
Loading control for Phostag BvrR gel 1
Fig 2
ChemiDoc Imaging System Bio-Rad

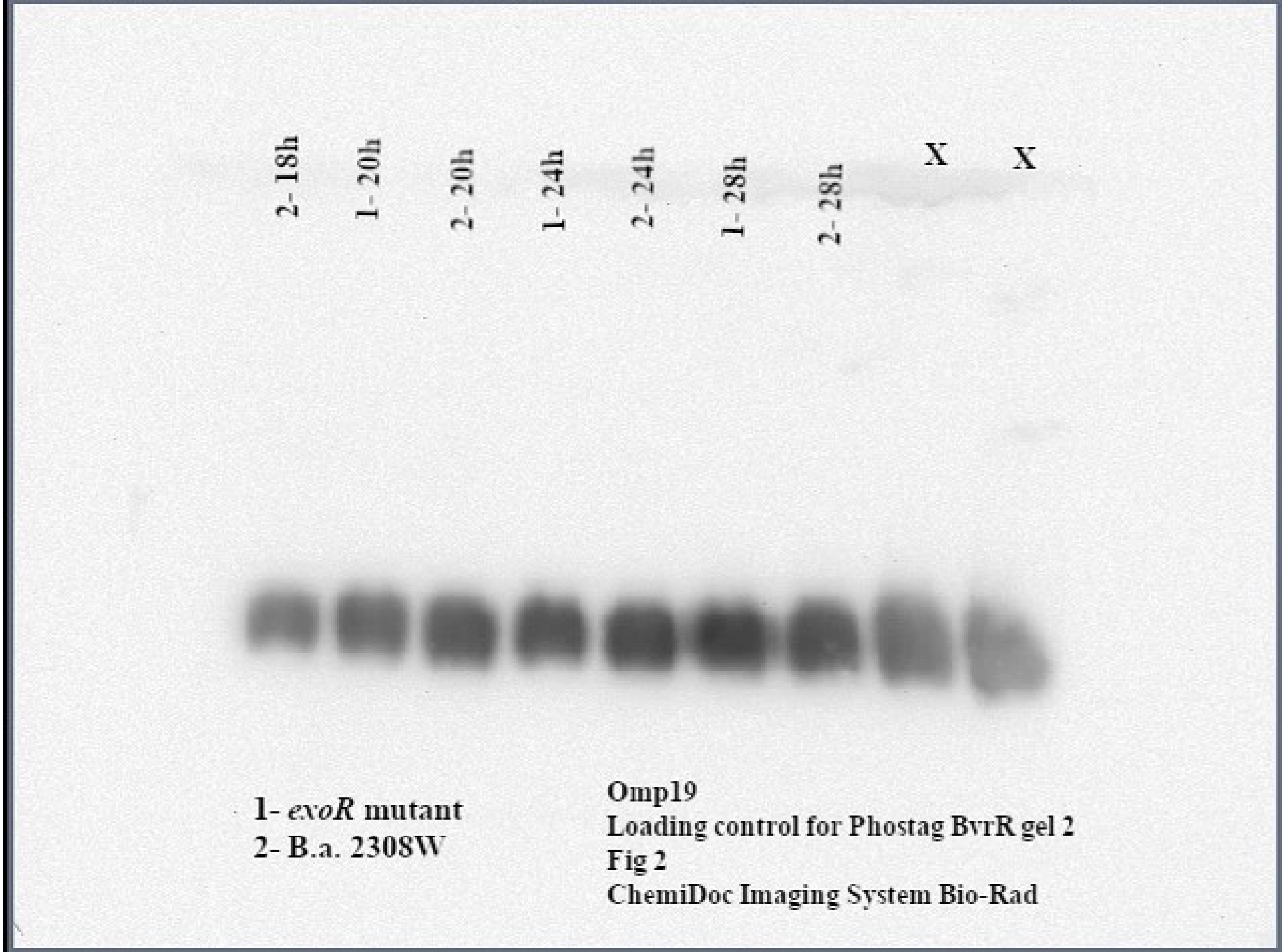
2- 18h
1- 20h
2- 20h
1- 24h
2- 24h
1- 28h
2- 28h
X
X
1- *exoR* mutant
2- B.a. 2308W
Omp19
Loading control for Phostag BvrR gel 2
Fig 2
ChemiDoc Imaging System Bio-Rad

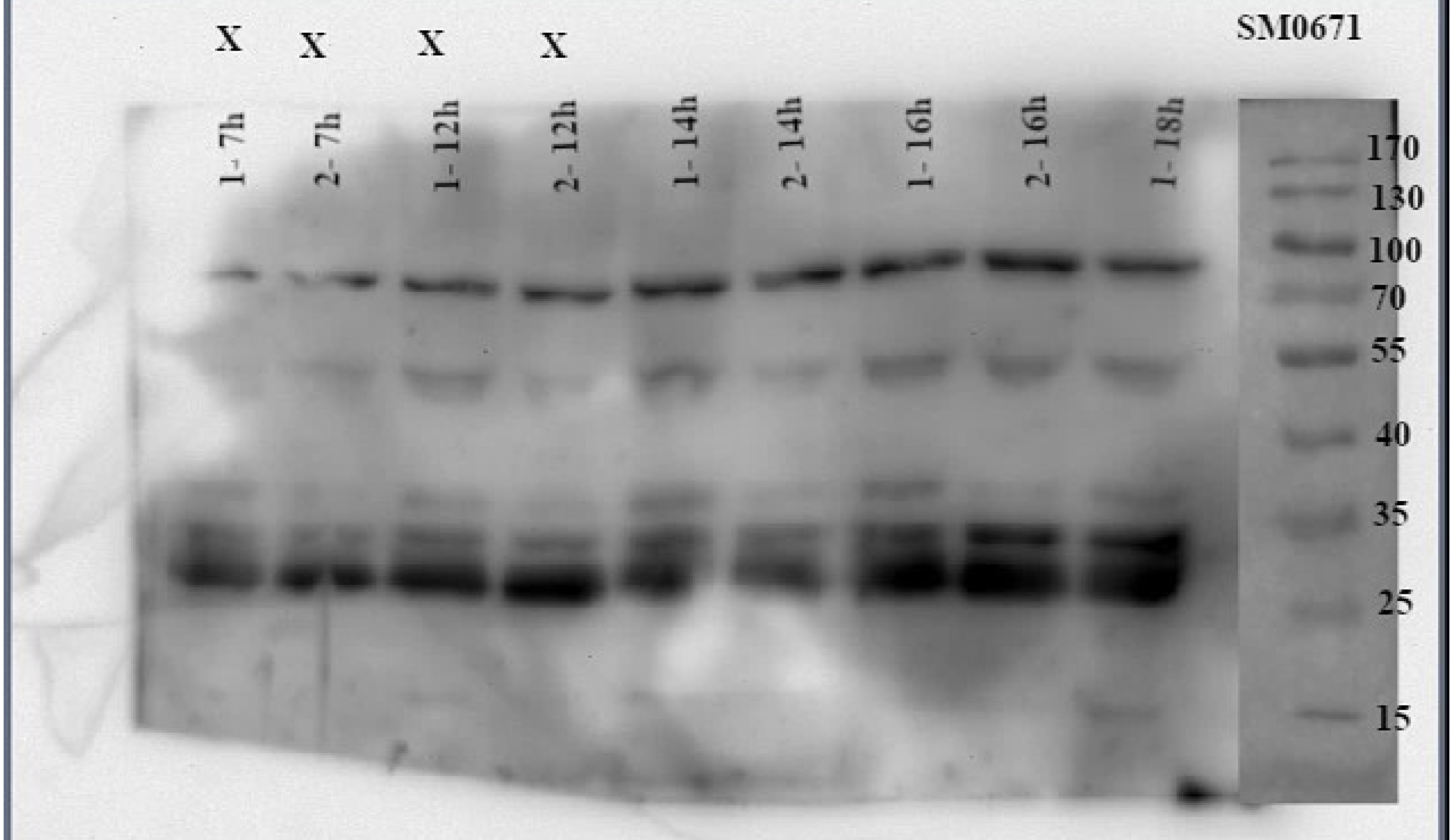

1- *exoR* mutant
2- B.a. 2308W

Fig 3 VjbR
ChemiDoc Imaging System Bio-Rad

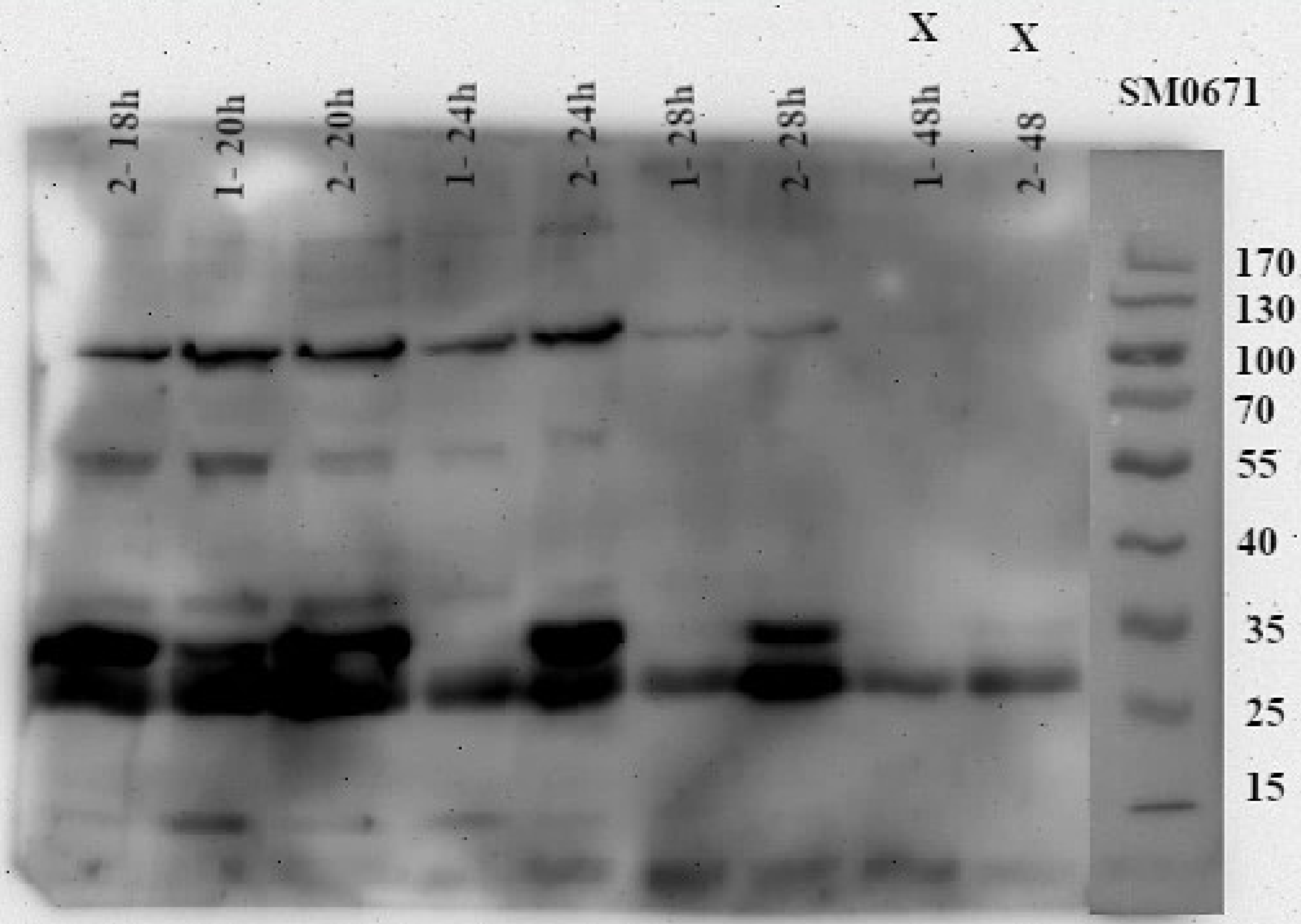

1- *exoR* mutant
2- B.a. 2308W

Fig 3 VjbR
ChemiDoc Imaging System Bio-Rad

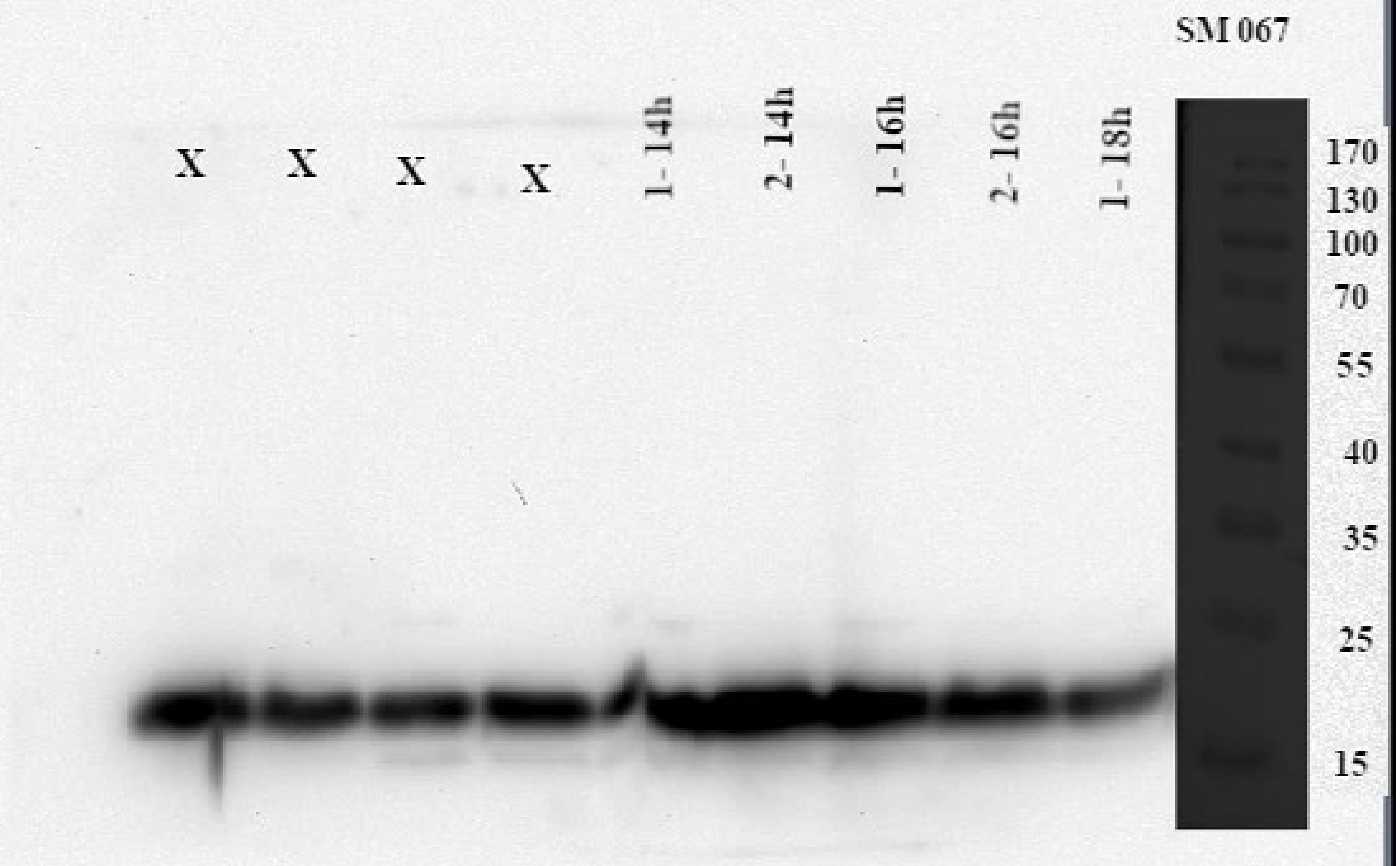

Omp19
Loading control for VjbR gel 19
Fig 3
ChemiDoc imaging system Bio-Rad

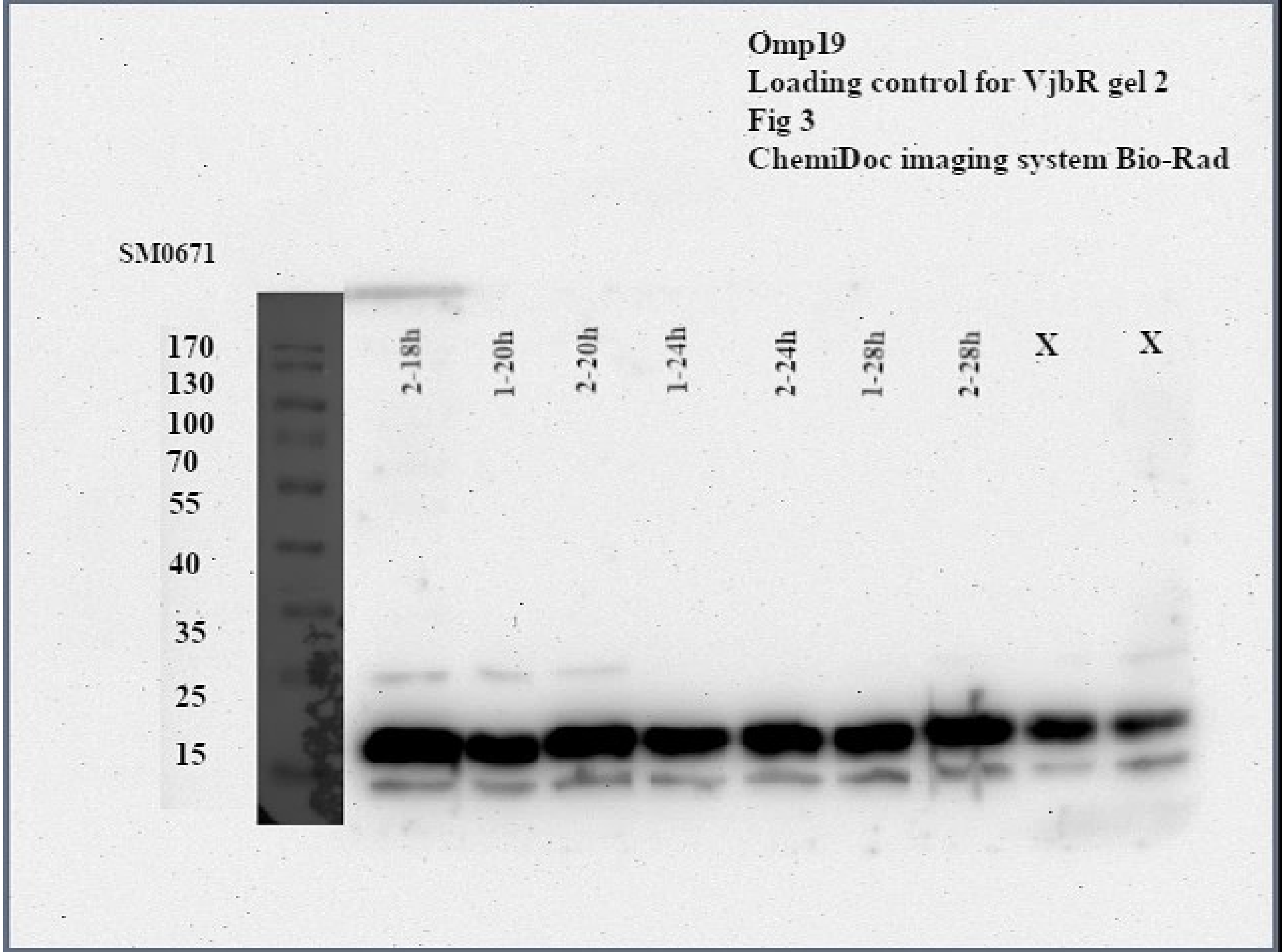
Omp19
Loading control for VjbR gel 2
Fig 3
ChemiDoc imaging system Bio-Rad
SM0671
170
130
100
70
55
40
35
25
15
2-18h
1-20h
2-20h
1-24h
2-24h
1-28h
2-28h
X
X

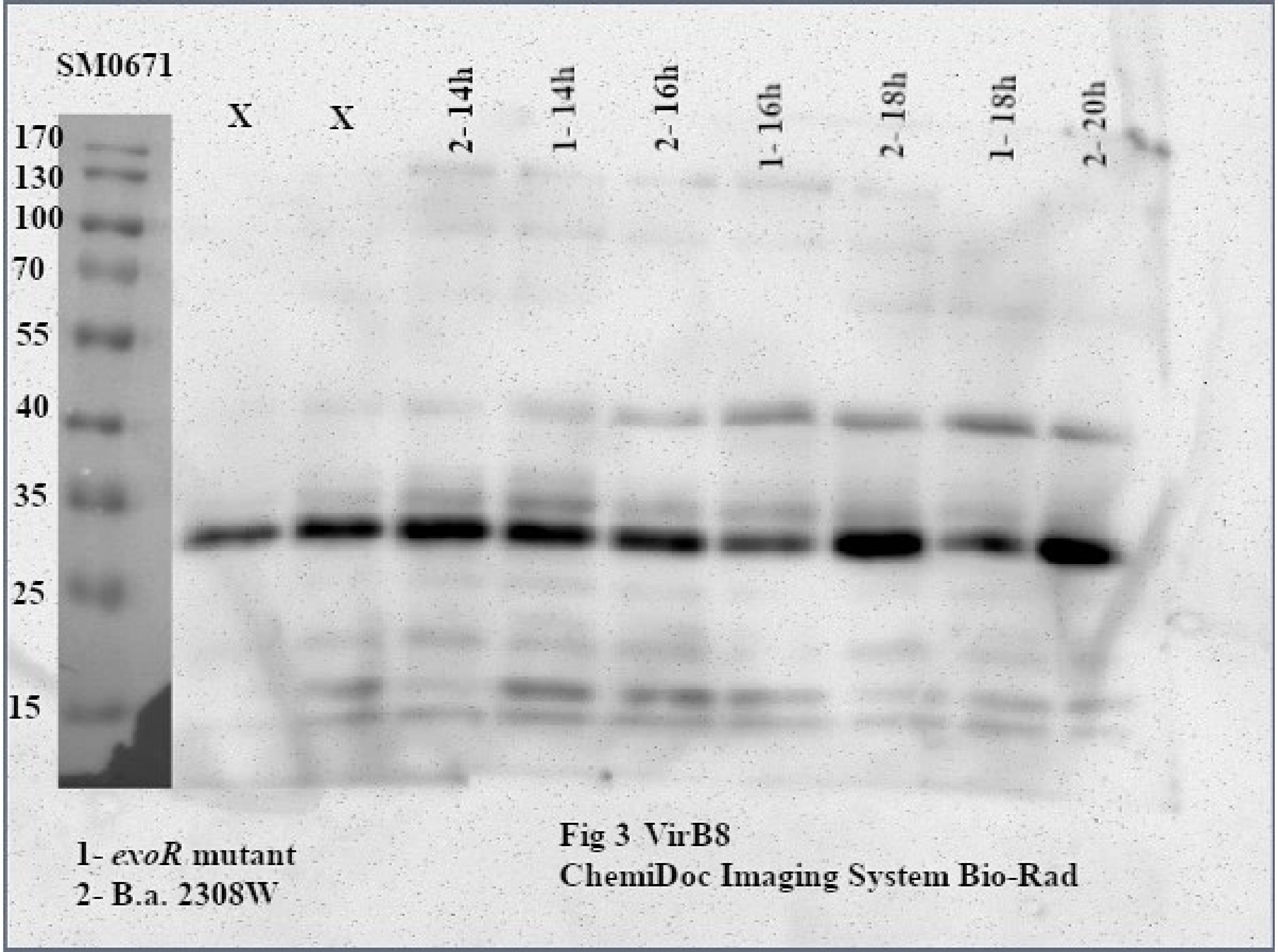

Fig 3 VirB8
ChemiDoc Imaging System Bio-Rad

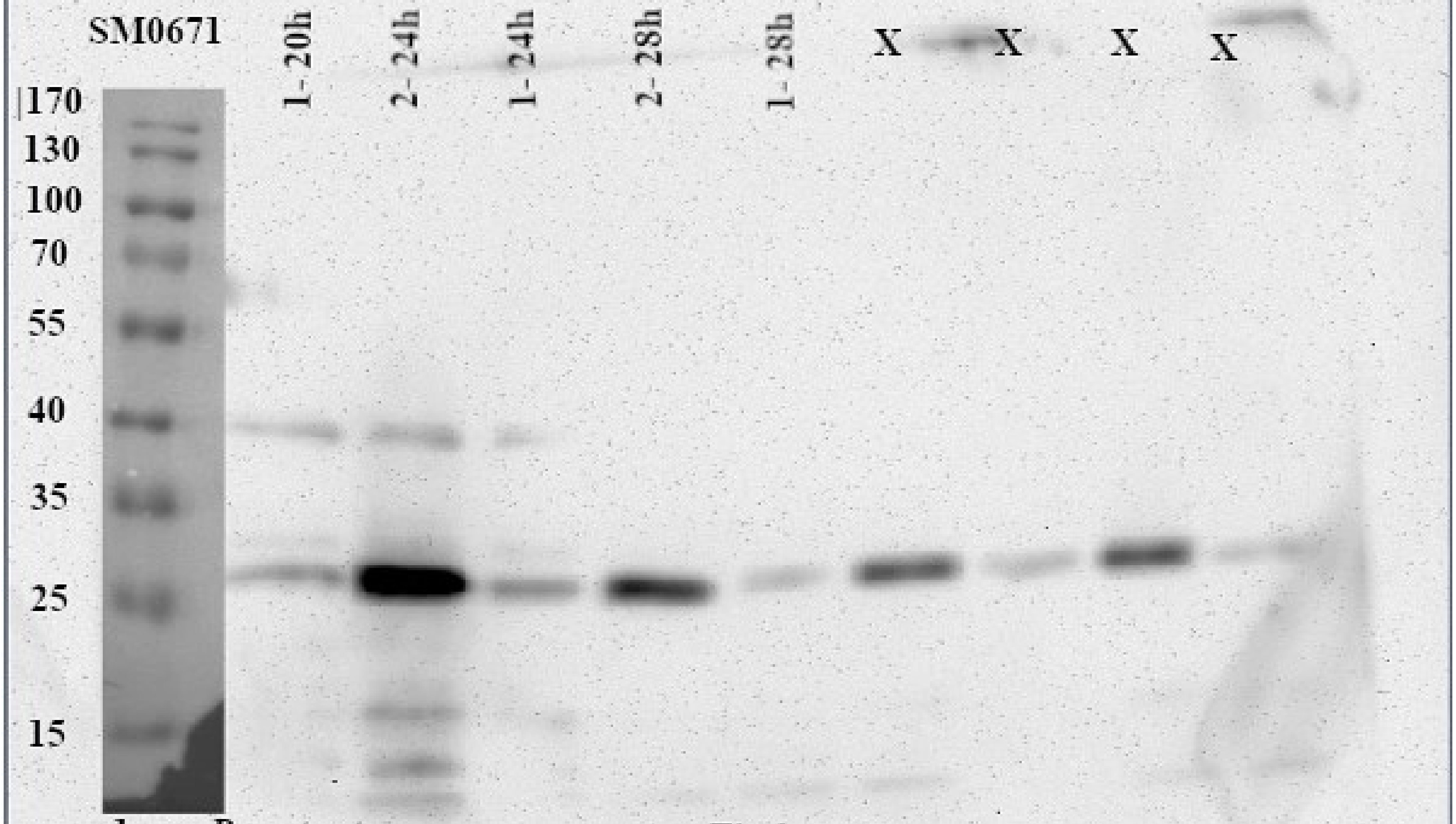

Fig 3 VirB8
ChemiDoc Imaging System Bio-Rad

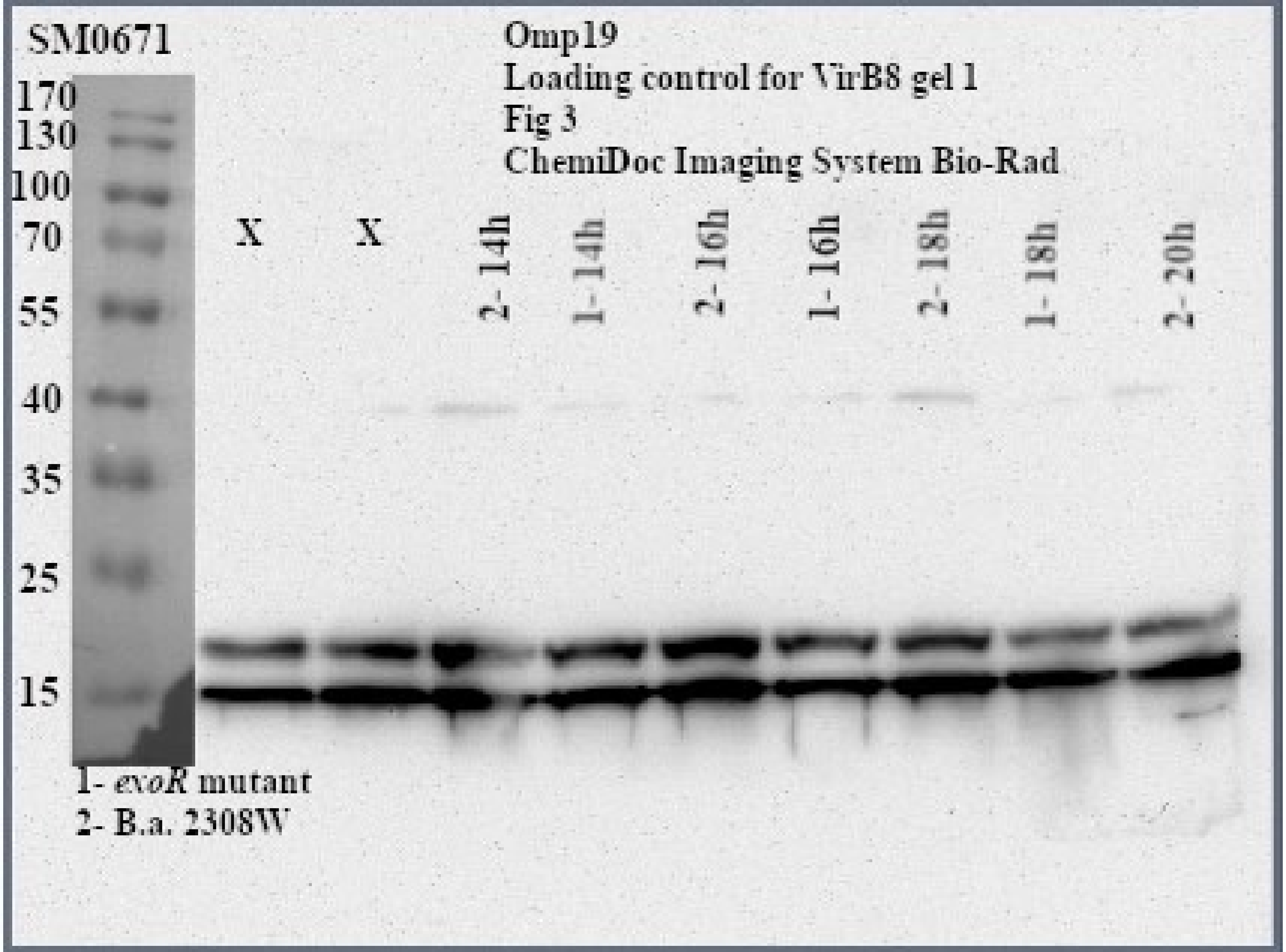
SM0671
Omp19
Loading control for VirB8 gel 1
Fig 3
ChemiDoc Imaging System Bio-Rad
170
130
100
70
55
40
35
25
15
X
X
2- 14h
1- 14h
2- 16h
1- 16h
2- 18h
1- 18h
2- 20h
1- *evoR* mutant
2- B.a. 2308W

1- *exoR* mutant
2- B.a. 2308W

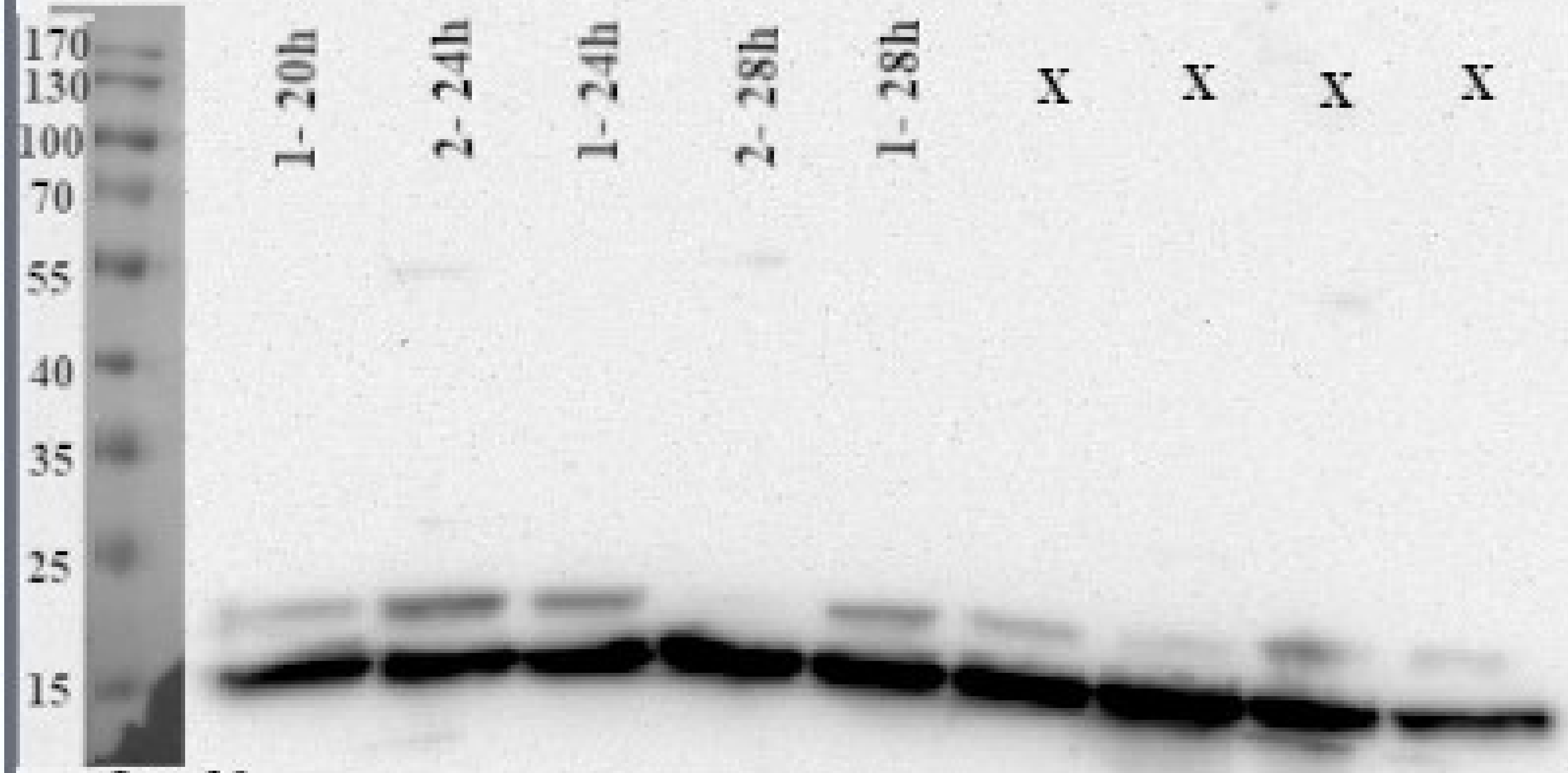

Omp19
Loading control for VirB8 gel 2
Fig 3
ChemiDoc Imaging System Bio-Rad

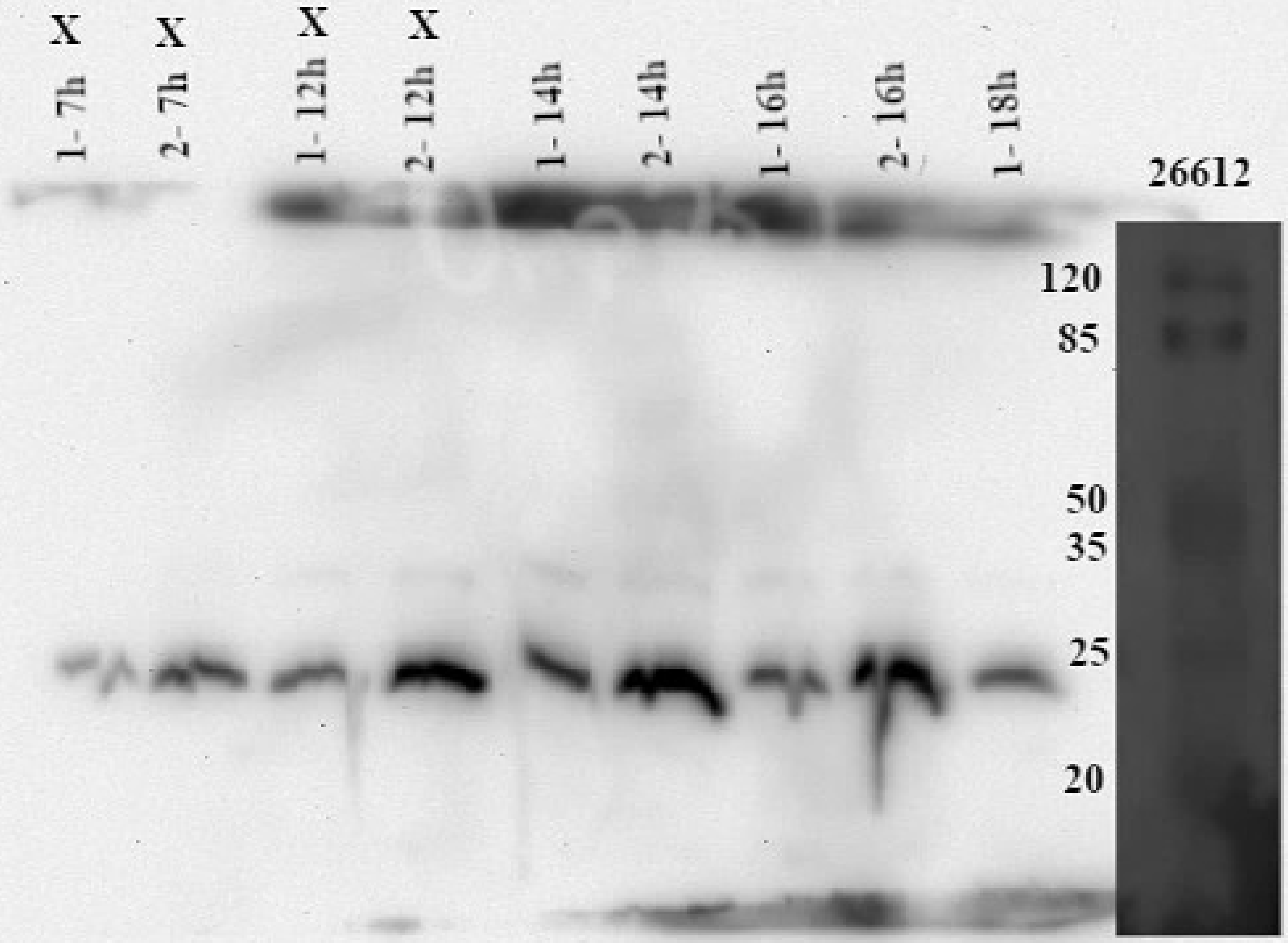

1- *exoR* mutant
2- B.a. 2308W

Fig 3 Omp25
ChemiDoc Imaging System Bio-Rad

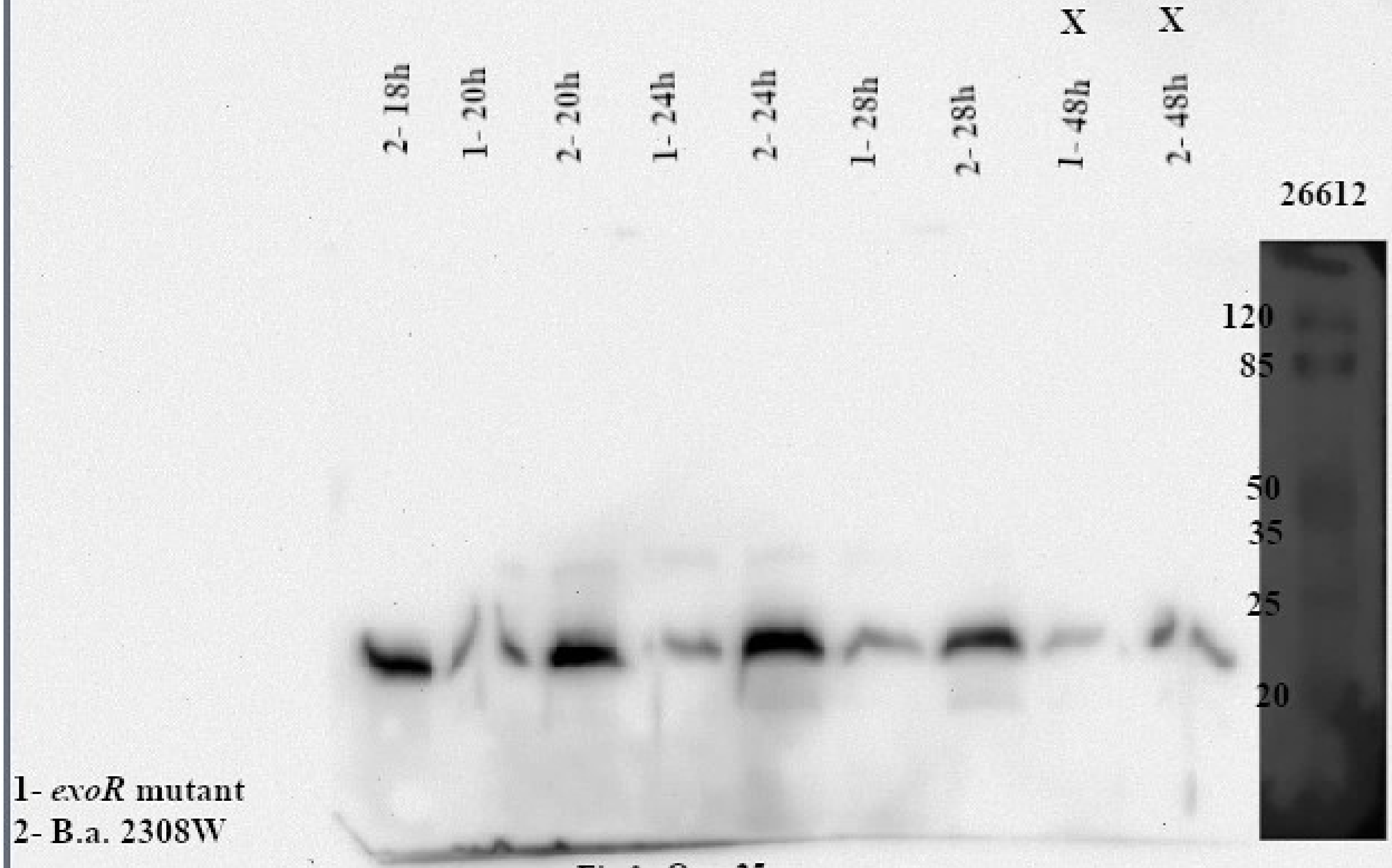

Fig 3 Omp25
ChemiDoc Imaging System Bio-Rad

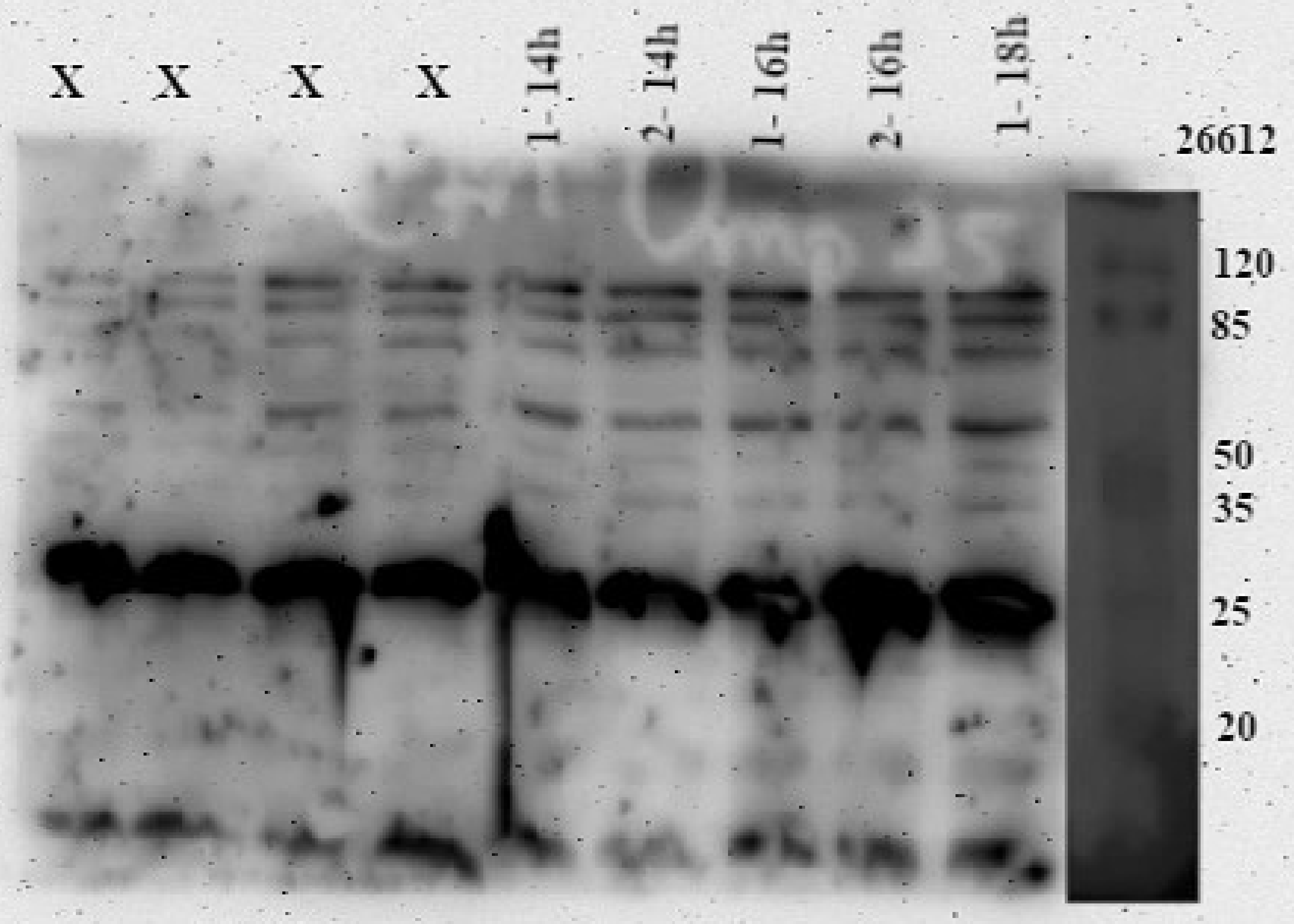

1- *exoR* mutant
2- B.a. 2308W

Bscp31
Loading control for Omp25 gel 1
Fig 3
ChemiDoc Imaging System Bio-Rad

Bscp31
Loading control for Omp25 gel 2
Fig 3
ChemiDoc Imaging System Bio-Rad

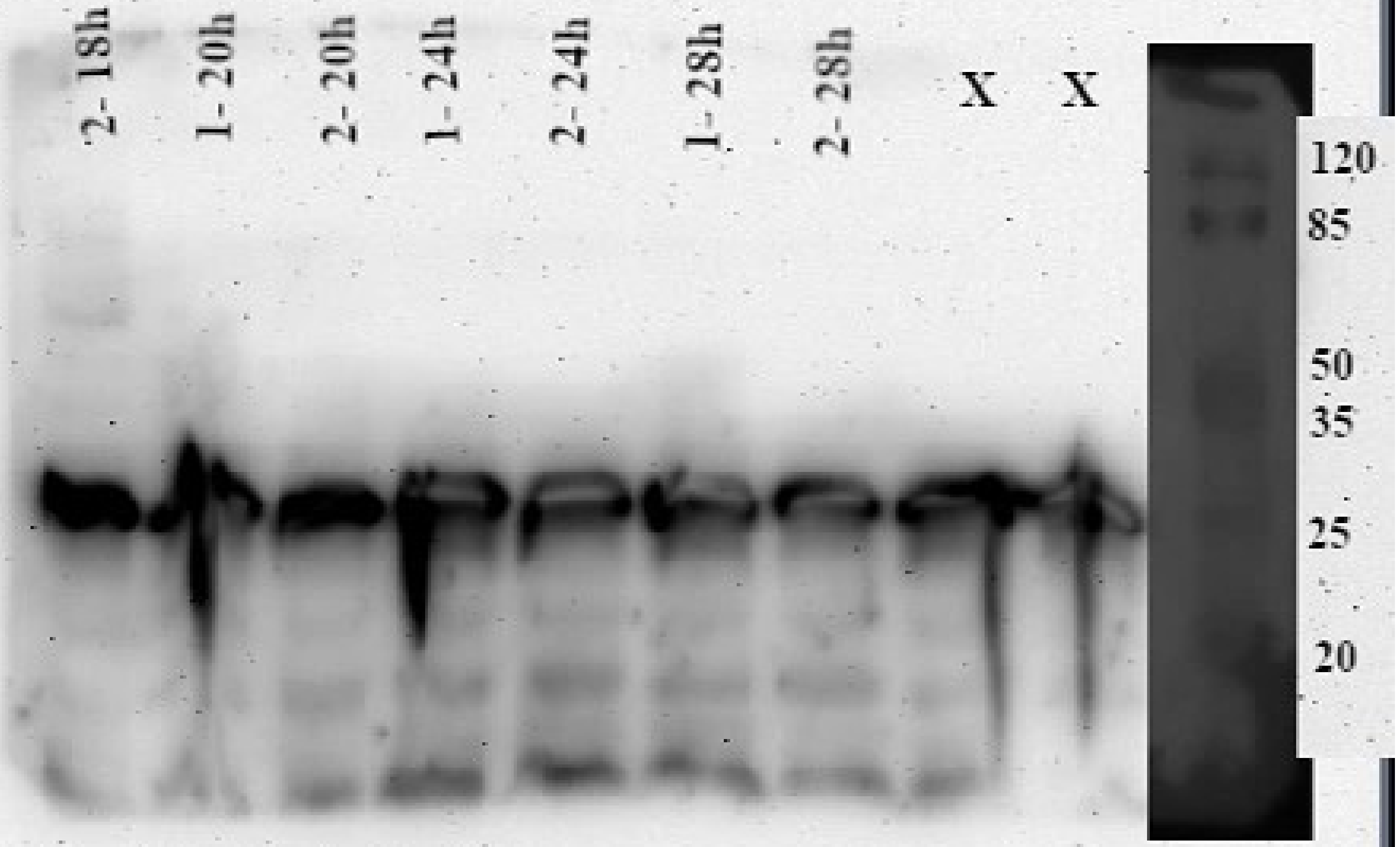

1- *evoR* mutant
2- B.a. 2308W

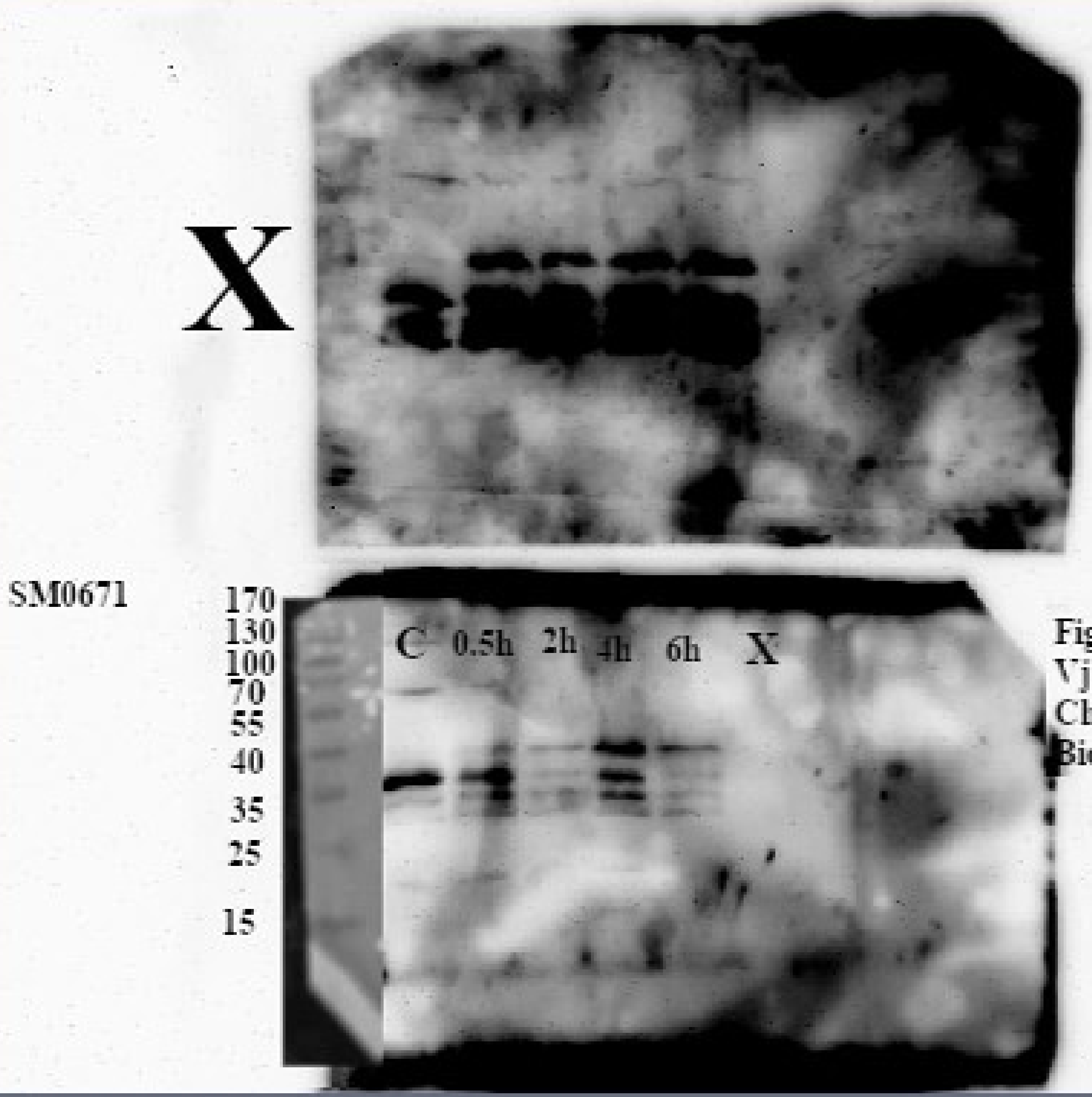

Fig4C
VjbR *exoR* mutant
Chemidoc Imaging System
Bio-Rad

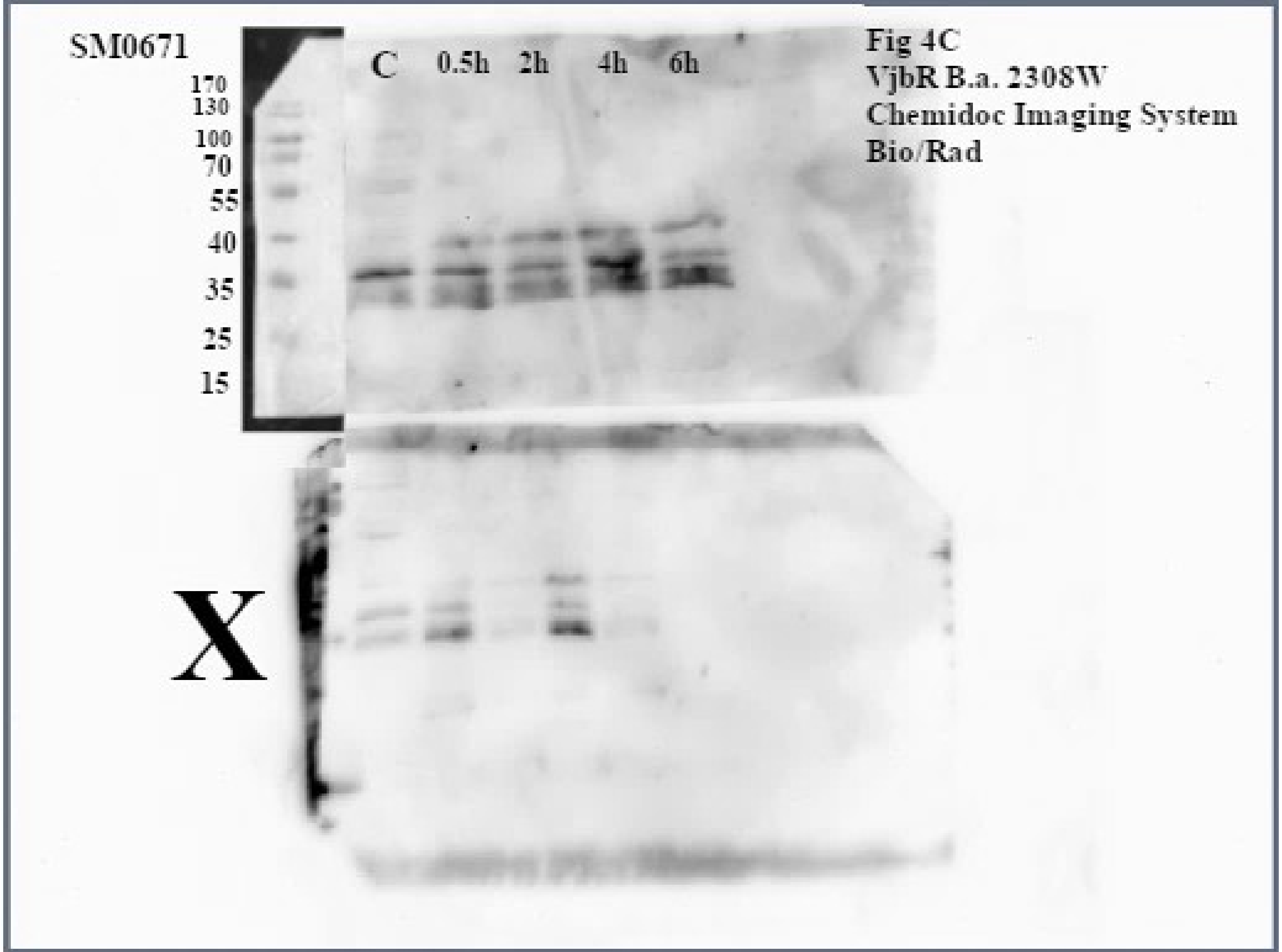
SM0671
170
130
100
70
55
40
35
25
15
C
0.5h
2h
4h
6h
Fig 4C
VjbR B.a. 2308W
Chemidoc Imaging System
Bio/Rad
X

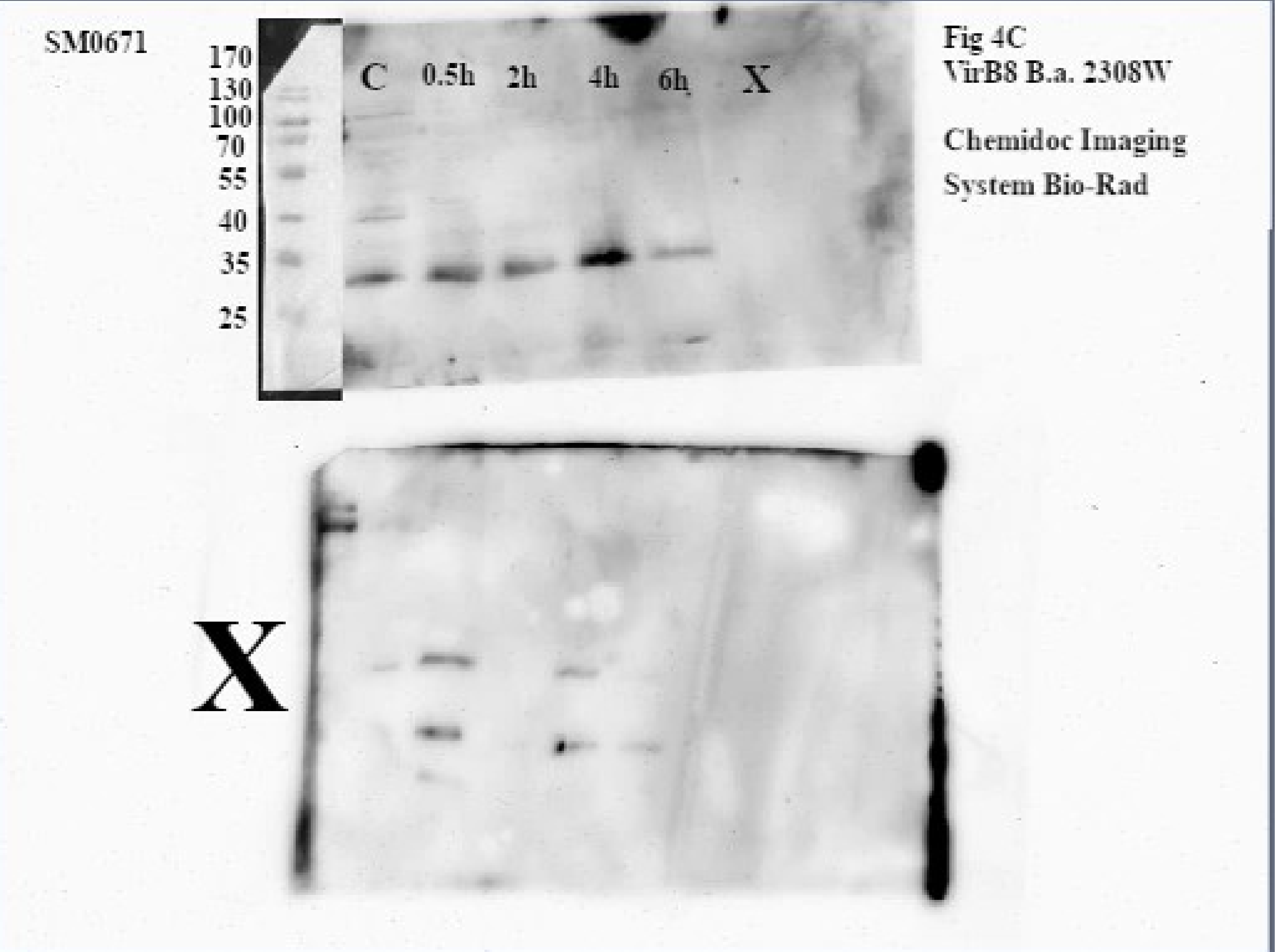

SM0671
170
130
100
70
55
40
35
25
C
0.5h
2h
4h
6h
X
Fig 4C
VirB8 B.a. 2308W
Chemidoc Imaging
System Bio-Rad
X

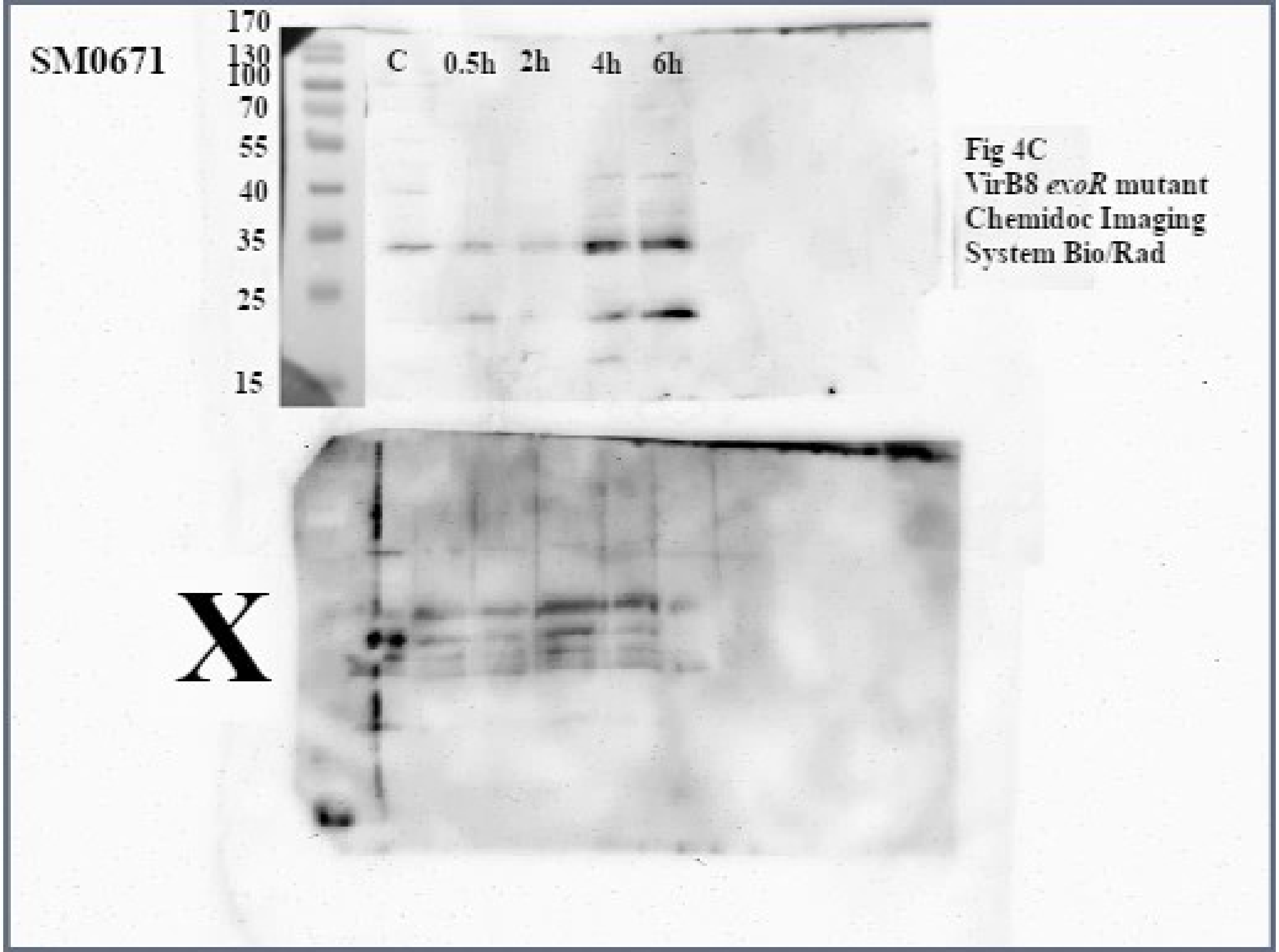
SM0671
170
130
100
70
55
40
35
25
15
C
0.5h
2h
4h
6h
Fig 4C
VirB8 *exoR* mutant
Chemidoc Imaging
System Bio/Rad
X

Fig 4C
Omp19 Loading control
Chemidoc Imaging System Bio-Rad

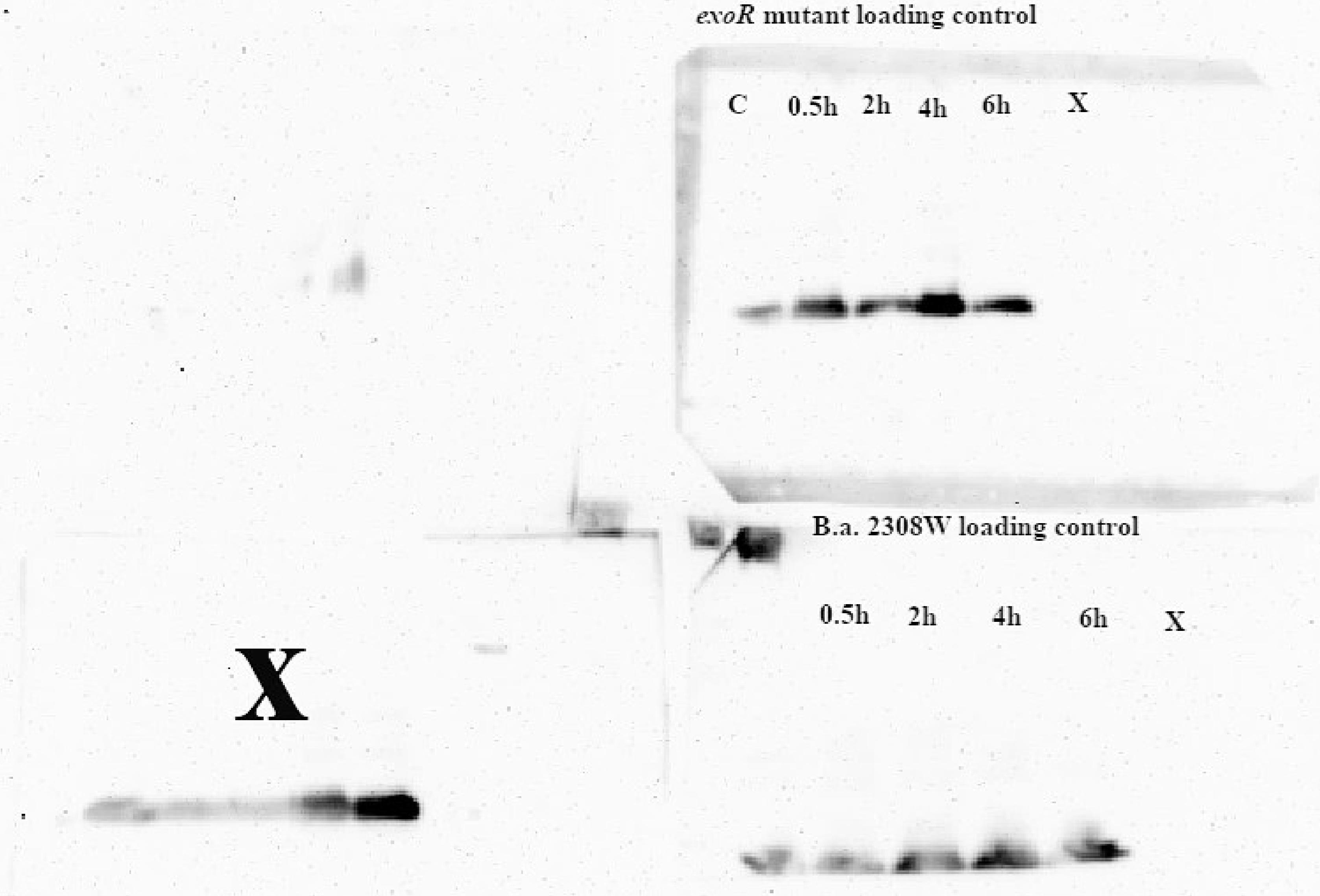

Supplement: S1 Raw images — (PDF) [file pone.0254568.s003.pdf]
